# Collagen Fingerprinting: A New Screening Technique for Radiocarbon Dating Ancient Bone

**DOI:** 10.1371/journal.pone.0150650

**Published:** 2016-03-03

**Authors:** Virginia L. Harvey, Victoria M. Egerton, Andrew T. Chamberlain, Phillip L. Manning, Michael Buckley

**Affiliations:** 1 School of Earth, Atmospheric and Environmental Sciences, The University of Manchester, Manchester, M13 9PL, United Kingdom; 2 Department of Geology and Environmental Geosciences, College of Charleston, 66 George Street, Charleston, South Carolina, 29424, United States of America; 3 Faculty of Life Sciences, The University of Manchester, Manchester M13 9PL, United Kingdom; University of Otago, NEW ZEALAND

## Abstract

Collagen is the dominant organic component of bone and is intimately locked within the hydroxyapatite structure of this ubiquitous biomaterial that dominates archaeological and palaeontological assemblages. Radiocarbon analysis of extracted collagen is one of the most common approaches to dating bone from late Pleistocene or Holocene deposits, but dating is relatively expensive compared to other biochemical techniques. Numerous analytical methods have previously been investigated for the purpose of screening out samples that are unlikely to yield reliable dates including histological analysis, UV-stimulated fluorescence and, most commonly, the measurement of percentage nitrogen (%N) and ratio of carbon to nitrogen (C:N). Here we propose the use of collagen fingerprinting (also known as Zooarchaeology by Mass Spectrometry, or ZooMS, when applied to species identification) as an alternative screening method for radiocarbon dating, due to its ability to provide information on collagen presence and quality, alongside species identification. The method was tested on a series of sub-fossil bone specimens from cave systems on Cayman Brac (Cayman Islands), chosen due to the observable range in diagenetic alteration, and in particular, the extent of mineralisation. Six ^14^C dates, of 18 initial attempts, were obtained from remains of extinct hutia, *Capromys* sp. (Rodentia; Capromyidae), recovered from five distinct caves on Cayman Brac, and ranging from 393 ± 25 to 1588 ± 26 radiocarbon years before present (yr BP). All of the bone samples that yielded radiocarbon dates generated excellent collagen fingerprints, and conversely those that gave poor fingerprints also failed dating. Additionally, two successfully fingerprinted bone samples were screened out from a set of 81. Both subsequently generated ^14^C dates, demonstrating successful utilisation of ZooMS as an alternative screening mechanism to identify bone samples that are suitable for ^14^C analysis.

## Introduction

Analyses of both extant and extinct fauna are essential for understanding the evolutionary ecology of discrete regions through time. The two most important pieces of information required are: 1) accurate species identification, and 2) an accurate chronological framework. For archaeological and palaeontological material the former of these is typically obtained through morphological analyses of the bones, whereas the latter can be achieved through various dating methods, but most accurately through the use of radiocarbon dating (when samples are sub ~50 k BP). Both of these sources of information are affected by environmental conditions, including climate (temperature and humidity changes), and other taphonomic considerations such as deposition environment, and matrix and pore water geochemistry. The tropics, which are most noted for high biodiversity, conversely have the poorest survival record for faunal remains due to the high temperature and humidity that adversely affects protein (i.e. collagen) survival, thereby reducing the probability of fossilisation [1]. Specimens that may appear [morphologically] to be relatively well preserved, often lack sufficient collagen yields for successful radiocarbon dating [2], resulting in either failed dating or the acquisition of an expensive date that is questionable due to potential external contaminant biomolecules [3]. Advanced methods of proteomic characterisation may therefore provide a better approach to investigate collagen preservation for successful and reliable dating, with the additional benefit of taxonomic identification.

### Current ^14^C screening techniques

The construction of a temporal framework of biodiversity patterns in faunal assemblages allows insight into the effects of ecological changes on habitats, including human-induced impacts. However, one of the most appropriate means to achieve such a framework—radiocarbon dating—is significantly costly, both in terms of equipment and time [4]. Radiocarbon dating is also destructive, especially when the specimens to be dated are small, and there are a number of commonly accepted issues that surround the success of this technique, including sample survival and contamination with external organics [5].

The degree of organic preservation remains a crucial concern when radiocarbon dating is applied to any bone material [6], and the preservation state depends on two main aspects; (i) time, and (ii) physical/biotic environment; or more likely, a combination of the two. Other than visual clues into diagenetic maturity, or external information relating to deposition environment (both of which can be highly uninformative), there are few simple and reliable sample selection procedures in the literature to screen bone appropriate for dating. Chiu *et al*. [7] used microscope observation to screen fossil corals in an attempt to avoid diagenetically-altered samples—a technique that also works well when observing microstructural qualities relating to diagenesis in bone tissue (e.g. [8, 9]). However, this technique is both time consuming and involves destruction of samples to obtain thin sections for light microscopy. Hoke *et al*. [10] suggested a more viable methodology, using UV-stimulated fluorescence to select samples with increased biomolecular preservation. Here, modern, fresh bone samples would display blue/white luminescence and degraded samples exhibited brown/yellow/grey fluorescence when a cross section was exposed to UV-light. Though simple, cheap and fairly reliable, this method is invasive and also requires samples to be destructively sectioned.

Laboratories that conduct ^14^C dating, such as the Oxford Radiocarbon Accelerator Unit (ORAU), occasionally measure the percentage of nitrogen (%N) in bone as a screening mechanism prior to dating [11]. As collagen deteriorates, %N decreases due to the relative decrease in amino acid content, and this relationship allows for a prediction of sample integrity. Investigating %N content is a rapid, simple and relatively cheap process and has been proven to have a success rate of up to 84% when applied with a %N threshold of 0.8 on well preserved, young bone samples. However, screening in older (i.e. Pleistocene, ~12 Ka– 2.5 Ma) bones resulted in a reduction in the reliability of predicted results to 68% [11]. Similarly, either the whole bone carbon:nitrogen atomic weight ratio (whole bone C:N) or the C:N ratio of purified collagen (collagen C:N) can be used to indicate the state of collagen preservation. A collagen C:N ratio of between 2.9 and 3.5 is widely utilised as an indicator of acceptable organic preservation for AMS radiocarbon dating and dietary stable isotope analysis [2, 3] and successful classification of samples into ‘well preserved’ or ‘poorly preserved’ categories has been shown to achieve a 71% success rate with a whole bone C:N threshold of 17.0 [11].

Routine collagen extraction procedures at radiocarbon laboratories often include ultrafiltration steps that work to improve ^14^C dates whilst removing some, but not all, exogenous contaminants (including organic acids) [12]. These contaminants from the depositional environment, particularly when coupled with low collagen content, have previously led to an acquisition of inaccurate, unreliable and expensive dates [12, 13]. Van Klinken [3] monitored various parameters, including percentage collagen yield (%yield), collagen C:N ratios and %N, in an attempt to measure levels of exogenous contamination in bone samples, though concluded that none were “sufficiently powerful to indicate contamination or degradation with sufficient sensitivity” ([3]: p.692). Independent techniques that test collagen presence and quality prior to ^14^C dating can therefore provide supporting evidence to ensure dates have been acquired from endogenous collagen rather than external contaminants, thereby producing reliable and publishable dates.

### Zooarchaeology by Mass Spectrometry

The collagen fingerprinting technique, often referred to as Zooarchaeology by Mass Spectrometry (ZooMS) [14], utilises small sample sizes (~1–50 mg; [15–18]) and can produce rapid results within 24–48 hours in a high-throughput manner at relatively low cost [18]; the success of which, much like radiometric dating, depends upon the detectable presence of Type I collagen (hereafter referred to as ‘collagen’) within the sample. Extracted collagen, analysed via soft ionisation mass spectrometry, relies upon a tryptic digestion to generate a peptide mass fingerprint (PMF). Exogenous contaminants that can skew dating should not affect a PMF to the same extent as dating. Additionally, collagen fingerprints are unique down to genus level in many organisms [19, 20], and in some cases to species level (e.g. [17, 21]), highlighting its worth in species identification and taxonomic partitioning. Collagen is known to survive longer than other genetically informative biomolecules, such as ancient DNA (aDNA), with successful fingerprinting from samples dating up to ~3.5 Ma [21]. Analysis of this sturdy triple-helical structural protein for species identification in this manner also allows circumvention of contamination issues that commonly occur through other techniques, such as polymerase chain reaction (PCR) required during aDNA amplification [14, 19, 22].

### Radiocarbon dating on the Cayman Islands

**Fig 1:**
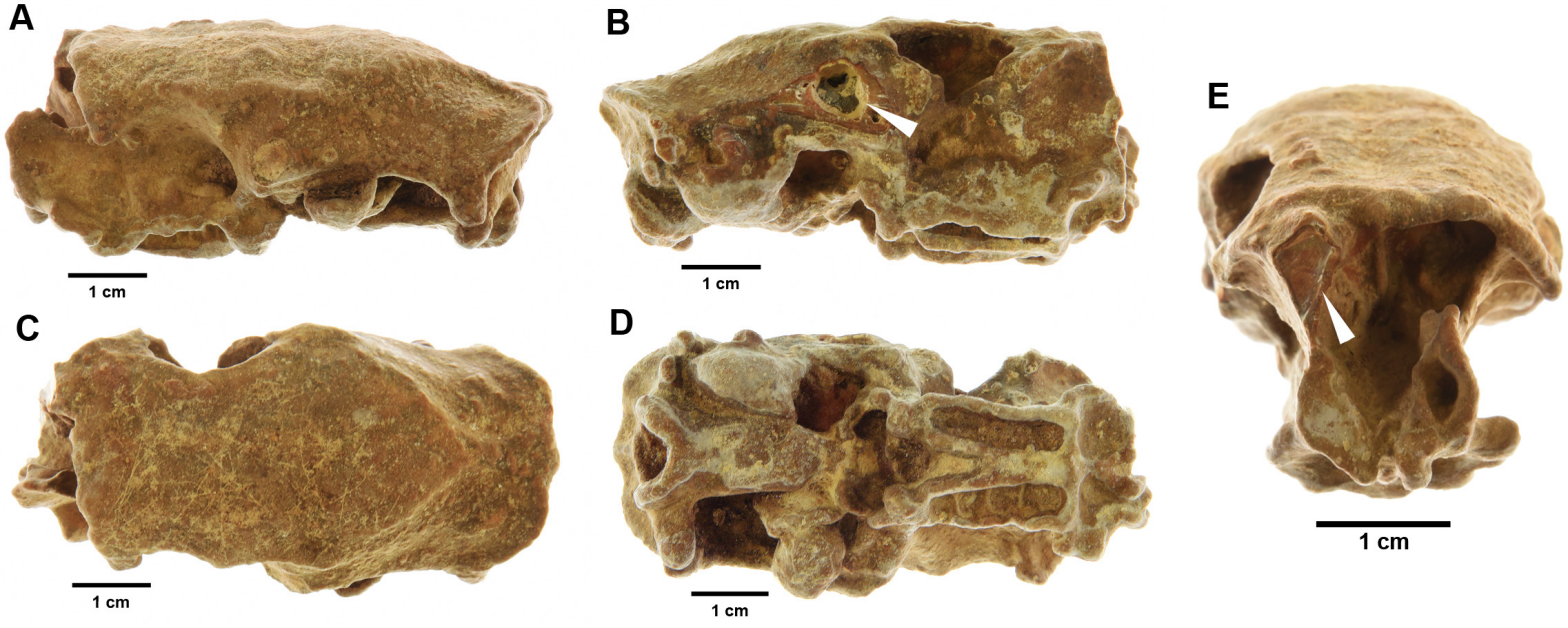
Skull from hutia, *Capromys* sp. (sample number 24, Bedding Plane II Cave, Cayman Brac) showing an example of the extent of mineralisation that is typical in Cayman Island assemblages. Images show left (A), right (B), dorsal (C), ventral (D) and anterior (E) sides of skull. For interest, arrows indicate sites where sampling was achieved for ^14^C dating and ZooMS analysis.

The best conditions for molecular preservation of Late Quaternary vertebrate remains in the tropics lies almost exclusively within cave systems [23]. Within such microenvironments, remains can become isolated from temperature changes, humidity variation, soil organisms and humic acids [24]. Bones deposited in caves may also become encased in calcareous matrices, such as is common on the Cayman Islands (Fig 1), increasing the likelihood of exceptional protein preservation. Samples such as these are ideal to perform ZooMS upon, given their potential for increased collagen survival.

The British Overseas Territory of Cayman Brac is a carbonate island with numerous caves and rock fissures that have naturally accumulated remains of island fauna over time. These caves act as natural repositories, providing potential data documenting island biodiversity through time. To date, only five radiocarbon (^14^C) dates have been obtained from the Cayman cave systems in an attempt to age the sub-fossils [25, 26] two from Grand Cayman and three from Cayman Brac (Patton’s Fissure, on the north coast of the island). On Grand Cayman ^14^C dating was conducted on the remains of Cuban crocodile, *Crocodylus rhombifer*, and on a femur of *Capromys* sp., yielding dates of 860 ± 50 yr BP and 375 ± 60 yr BP respectively [26]. Additionally, ^14^C dates from Cayman Brac have been generated from land snail shell samples from three distinct substrate layers in Patton’s Fissure: 80–100 cm below the surface of the sediments, 11,180 ± 105 yr BP; 120–140 cm below surface, 13,230 ± 135 yr BP; and 160 cm below surface, 13,850 ± 135 yr BP [25]. These dates should be considered maximum ages due to the constraints associated with radiocarbon dating snail shells which may contain some geologically old carbon [27], however, the oldest fossils are thought likely to be at least late Pleistocene in age [26].

ZooMS and ^14^C dating can be utilised to unlock both temporal fidelity and species identification within cave deposits, such as those observed on Cayman Brac. In this paper, we investigate the feasibility of using a rapid technique of ZooMS to identify samples that are suitable for ^14^C dating. Such an application is particularly advantageous for localities in which collagen survival may be variable between or even within sites (i.e. in the tropics). This consolidated ZooMS methodology requires no more, and often less, destruction than sampling for radiocarbon dating would require. We utilise this technique to qualitatively assess the degree of collagen preservation within sub-fossil mammal bone collected from Cayman Brac (Cayman Islands) and thus apply it as a screening procedure prior to ^14^C dating. There are two key advantages of ZooMS collagen fingerprinting in this respect: 1) ZooMS offers faunal identification, which is particularly useful in sites that host limited and/or highly fragmented material, or intentionally modified material such as bone tools, and 2) ZooMS screening helps circumvent the acquisition of dates from external contaminants rather than bone collagen by confirming the quality of the surviving collagen.

## Materials and Methods

The specimens collected in this study are permanently reposited in the departmental collection of the School of Earth, Atmospheric & Environmental Sciences (University of Manchester, Manchester, UK) within the Cayman Brac (CB) sub-fossil collection and are accessible to all researchers. Specimens 3–25 were labelled MB3-25 on initial submission for radiocarbon dating, with later samples 430 and 433 dated under the labels MB29 and MB30 respectively. No permits were required for the described study, which complied with all relevant regulations working in conjunction with the Department of Environment (Cayman Islands). The Cayman Islands’ legal system is based on English common law, locally enacted statutes and Orders-in-Council. Local principal statutes (laws) are passed by the Legislative Assembly and assented to by the Governor. The cave deposits studied and collected from the Cayman Islands are currently not protected by any local laws. Locality and horizon data are described below.

### Sampling Locations

The Cayman Islands comprise three small, low-lying subtropical islands located in the north-west of the Caribbean Sea, all of which are considered to be geographically part of the Greater Antilles and of the West Indies (Grand Cayman: 19°21N, 81°17W; Little Cayman: 19°43N, 80°03W; Cayman Brac: 19°43N, 79°51W). Little Cayman and Cayman Brac lie within 7 km of one another, and are located about 130 km north-west of Grand Cayman.

Extensive cave systems occur throughout the Cayman Islands, but they are most accessible on Cayman Brac. Vertebrate sub-fossil samples were collected from five distinct caves: Green Cave (19°43'9.12"N, 79°46'4.02"W), Pebble Cave (19°43'7.13"N, 79°46'12.69"W), Shelby’s Bolt Hole (19°44'01.36"N, 79°46'45.76"W), Bedding Plane Cave I (19°44'7.73"N, 79°44'15.64"W) and Bedding Plane Cave II (19°44'6.82"N, 79°44'16.71"W). A total of twenty bones were radiocarbon dated in this study (Table 1). Sample numbers 3 to 25 (n = 18) were chosen due to a thick covering of geological matrix, given such specimens may be better preserved and better protected from contamination. A further 81 specimens, not previously radiocarbon dated, were also screened from a range of chambers within Green Cave, including five stratigraphic layers within a pilot excavation (Table 2). Of these 81 samples, a sub-selection of two samples (430 and 433) were sent for subsequent ^14^C dating. These two samples were selected due to the quality of their collagen fingerprints, larger size and deposition location deep within Green Cave (Chamber 5; see Table 2).

**Table 1:** Deposition location, %yield, %C, collagen C:N ratios, ^14^C dates, calibrated calendar dates (AD, 95% confidence range) and ZooMS result of deposited bone samples (n = 20) from five caves on Cayman Brac.

Sample Number	Cave Name	Cave Chamber	Skeletal Element	%yield	%C	C:N	δ^13^C (AMS)	^14^C age (yr BP)	cal. AD	ZooMS Result
3	Green Cave	2	Femur	X	X	X	X	X	X	X
4	Green Cave	2	Mandible	13.0	40.7	3.249	-18.32	609 ± 26	1296–1404	✓
5	Green Cave	3	Pelvis	X	X	X	X	X	X	X
6	Green Cave	3	Mandible	7.7	42.9	3.288	-18.35	928 ± 26	1031–1162	✓
7	Green Cave	4	Humerus	1.3	41.8	3.322	-17.59	1588 ± 26	411–540	✓
8	Green Cave	4	Pelvis	X	X	X	X	X	X	X
10	Green Cave	5, Surface	Tibia	X	X	X	X	X	X	X
11	Green Cave	5, Context 2	Femur	X	X	X	X	X	X	X
14	Green Cave	5, Context 3	Pelvis	X	X	X	X	X	X	X
15	Green Cave	5, Context 4	Indeterminate	X	X	X	X	X	X	X
17	Pebble Cave	Front	Indeterminate	X	X	X	X	X	X	X
18	Pebble Cave	Back	Femur	9.1	42.1	3.296	-19.03	393 ± 25	1440–1522, 1575–1624	✓
19	Shelby's Bolt Hole	Front	Femur	X	X	X	X	X	X	X
20	Shelby's Bolt Hole	Back	Femur	X	X	X	X	X	X	X
22	Bedding Plane I	n/a	Sacrum	X	X	X	X	X	X	X
23	Bedding Plane II	Entrance	Tibia	4.9	42.5	3.379	-19.23	897 ± 23	1042–1105, 1117–1210	✓
24	Bedding Plane II	Back	Skull	X	X	X	X	X	X	X
25	Bedding Plane II	n/a	Femur	8.0	42.6	3.341	-19.54	930 ± 25	1031–1160	✓
430*	Green Cave	5, Surface	Vertebra	8.1	43.3	3.23	-17.69	1134 ± 34	777–793, 802–987	✓
433*	Green Cave	5, Surface	Long bone—Indeterminate	9.2	43.2	3.231	-18.09	1166 ± 34	771–969	✓

✓ = positive result;

X = failed result.

A small pit was excavated in Chamber 5 of Green Cave and samples removed from the following contexts: 2 = 15–20 cm; 3 = 20–25 cm; 4 = 25–35 cm in depth.

*Dates for samples 430 and 433 were achieved following ZooMS pre-screening, where they were selected from a set of 81 bones (see also Table 2).

**Table 2:** A test for ZooMS as a pre-screening method, showing deposition location and ZooMS result for 81 deposited bone samples from Chamber 5, Green Cave (Cayman Brac).

Sample code	Deposition Location in Green Cave	Skeletal Element	ZooMS Result
143	Surface	Phalanx	X
421	Surface	Vertebra	X
424	Surface	Rib	✓
425	Surface	Rib	✓
458	Surface	Long bone (indeterminate)	✓
146	Surface	Indeterminate	X
422	Surface	Long bone (indeterminate)	X
423	Surface	Long bone (indeterminate)	X
430*	Surface	Vertebra (partial)	✓
459	Surface	Vertebra	X
460	Surface	Vertebra	X
159	Surface	Vertebra	✓
431	Surface	Long bone (indeterminate)	X
432	Surface	Long bone (indeterminate)	X
433*	Surface	Long bone (indeterminate)	✓
158	Surface	Rib (partial)	X
429	Surface	Rib	✓
461	Surface	Rib (partial)	X
149	Surface	Phalanx	X
150	Surface	Phalanx	X
154	Surface	Phalanx	X
155	Surface	Phalanx	X
428	Surface	Phalanx	X
145	Surface	Rib (partial)	X
467	Context 2	Long bone (indeterminate)	X
463	Context 2	Femur	X
464	Context 2	Femur	X
256	Context 2	Long bone (indeterminate)	X
434	Context 2	Long bone (indeterminate)	X
466	Context 2	Long bone (indeterminate)	✓
465	Context 2	Long bone (indeterminate)	X
438	Context 2	Long bone (indeterminate)	X
220	Context 2	Phalanx	X
221	Context 2	Phalanx	X
222	Context 2	Phalanx	X
435	Context 2	Phalanx	X
437	Context 2	Indeterminate	X
211	Context 2	Vertebra	X
212	Context 2	Vertebra	X
436	Context 2	Vertebra	X
462	Context 2	Vertebra	X
203	Context 2	Rib (partial)	X
204	Context 2	Rib (partial)	X
205	Context 2	Rib (partial)	X
206	Context 2	Rib (partial)	X
207	Context 2	Rib (partial)	X
266	Context 3	Phalanx	X
267	Context 3	Phalanx	X
268	Context 3	Phalanx	X
269	Context 3	Phalanx	X
270	Context 3	Phalanx	X
472	Context 3	Indeterminate	X
470	Context 3	Humerus	X
471	Context 3	Humerus	X
439	Context 3	Vertebra	X
440	Context 3	Vertebra	X
441	Context 3	Long bone (indeterminate)	X
442	Context 3	Long bone (indeterminate)	X
468	Context 3	Femur	X
469	Context 3	Femur	X
359	Context 3	Indeterminate	X
360	Context 3	Indeterminate	X
361	Context 3	Indeterminate	X
362	Context 3	Indeterminate	X
363	Context 3	Indeterminate	X
364	Context 3	Indeterminate	X
443	Context 3	Indeterminate	X
406	Context 4	Skull (partial)	X
476	Context 4	Vertebra (partial)	X
477	Context 4	Vertebra (partial)	X
479	Context 4	Skull (partial)	X
481	Context 4	Phalanx	X
409	Context 4	Long bone (indeterminate)	X
410	Context 4	Long bone (indeterminate)	X
480	Context 4	Long bone (indeterminate)	X
407	Context 4	Indeterminate	X
408	Context 4	Indeterminate	X
478	Context 4	Indeterminate	X
473	Context 4	Long bone (indeterminate)	X
474	Context 4	Long bone (indeterminate)	X
475	Context 4	Long bone (indeterminate)	X

Bone were deposited across a range of contexts: 2 = 15–20 cm; 3 = 20–25 cm; 4 = 25–35 cm in depth.

✓ = positive result;

X = failed result.

*Samples 430 and 433 were sent to ORAU for ^14^C dating following ZooMS pre-screening (see also Table 1).

### Methods of Analysis

#### Radiocarbon (^14^C) Dating

Samples were dated using Accelerator Mass Spectrometry (AMS) at the Oxford Radiocarbon Accelerator Unit (ORAU), using a standard protocol [28–30]. Geological matrix encasing each sample was mechanically removed from each of the eighteen samples, and bone powder obtained. Each sample was tested for percentage collagen yield (%yield) of which those indicating <1% of the starting weight of bone powder were automatically failed. Samples with sufficient %yield were subjected to a standard chemical pretreatment, target preparation and AMS measurement where approximately 0.5–1 g of the powder was loaded into a continuous-flow cell. Over a period of 8 h, an automated sequence of acid, base, acid (ABA) flow was applied to each sample, rinsing with ultrapure (MilliQ^™^) water between each reagent. Crude collagen was gelatinised by heating at 75°C in 1 mM hydrochloric acid (HCl) solution (pH 3.0) for 20 h, filtered using a 100 μL polyethylene Eezi-filter^™^ and the acid-insoluble pellets discarded. Supernatant was ultrafiltered (Vivaspin^™^ 15 30 kDa molecular weight cut-off; MWCO) and centrifuged (2500–3000 rpm) until 0.5–1 mL of the fraction remained. Resulting purified gelatin was freeze-dried and combusted in a CHN analyser, with separation of peptides >30 kDa. Results were applied to calibration plots using Oxcal computer programme (v4.2) of C. Bronk Ramsey, using the ‘IntCal13’ dataset as described by Ramsey *et al*. [31].

#### Zooarchaeology by Mass Spectrometry (ZooMS)

Materials and chemicals were purchased as in Buckley *et al*. [20]: HCl, acetonitrile (ACN) and sequencing-grade trypsin were obtained from Merck (UK), Fisher Scientific (UK) and Promega (UK) respectively. Trifluoroacetic acid (TFA), ammonium bicarbonate (ABC), mass spectrometric standards (calibration peptides) and α-cyano-4-hydroxycinnamic acid (matrix solution) were procured from Sigma-Aldrich (UK).

Collagen peptide fingerprints were obtained following methods modified from Buckley *et al*. [14] and van der Sluis *et al*. [17] utilising acid-soluble collagen. Sub-fossil bone samples from Cayman Brac were drilled using a diamond-tipped Dremel™ drill, in the same locality as the initial sample for ^14^C dating was undertaken, to avoid issues caused by variability in preservation within each sample. Approximately 5 mg of matrix-free bone powder was acquired. Bone powder was subsequently demineralised in 1 mL 0.6 M HCl over 3 h at room temperature. Bone samples from the skeletal remains of known reference species, *Capromys pilorides* (GH28/14; Cuban), were demineralised, as whole fragments or as drilled samples (required for samples of large size), in 1 mL 0.6 M HCl for 18 h at 4°C. Samples were then centrifuged for 5 min at 14,000 x *g*, and supernatant filtered using 10 kDa MWCO ultrafilters (Vivaspin, UK) and spun at 14,000 x *g* for 30 min. The ultrafilter supernatant for each sample was discarded and the retained collagen washed twice with 0.5 mL 50 mM ABC and centrifuged at 14,000 x *g* for each wash. The acid-soluble collagen was then resuspended using 100 μl 50 mM ABC and digested with 0.4 μg trypsin (Promega, UK) for 18 hr at 37°C in a separate Eppendorf tube.

Sample peptide mixtures were then purified (peptides desalted and concentrated) using C18 solid phase extraction pipette tips (Varian, UK), through methodology presented by Buckley *et al*. [14] and van der Sluis *et al*. [17], to allow peptide separation and facilitate species identification. Here, chosen samples were acidified to 0.1% TFA and fractioned. Fractions were dried and resuspended using 10 μL 0.1% TFA, and 1 μL spotted onto the Bruker target plate with 1 μL of a-cyano-4-hydroxycinnamic acid matrix solution per spot (0.1% TFA in ACN/H_2_O 1:1 v/v), and air-dried. Mass spectra from the collagen digest fractions were obtained using a Bruker Ultraflex II MALDI mass spectrometer (MS) operating at up to 5000 laser shots per plate spot, over a *m/z* range of 700–3700, and calibrated against an adjacent MS standard spot containing five calibrant peptides of 0.9 to 3.7 kiloDalton range (des-Arg^1^-bradykinin, angiotensin I, Glu^1^-fibrinopeptide, ACTH 1–17 clip, ACTH 18–39 clip and ACTH 7–38 clip). Mass peptide fingerprints were analysed using mMass software (v5.5.0) and peaks were filtered with a signal-to-noise (S/N) threshold of 5.0. Spectra were deemed successful when the number of labelled peaks above *m/z* 1500 was >10 in the 10% fractionation, following the S/N filter, to alleviate false positive results caused by matrix peaks or weak collagen signals.

## Results

**Fig 2:**
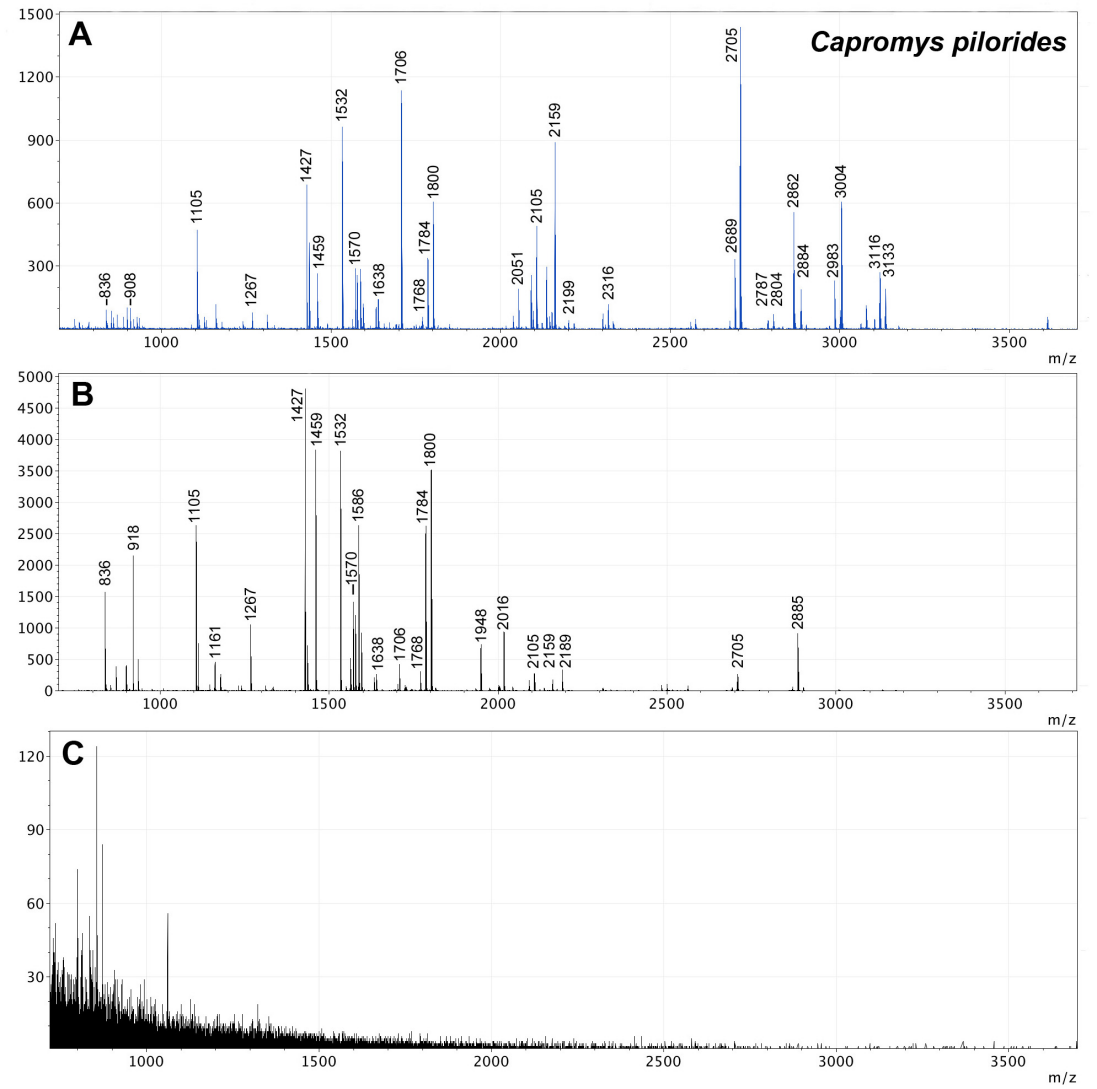
Example MALDI-MS spectra showing peptide mass fingerprints (PMFs) from: (A) collagen extracted from a reference sample of *Capromys pilorides* following digestion with trypsin; (B) 10% ACN fractionation of Cayman Brac sub-fossil sample number 7 following digestion with trypsin and purification using C18 solid phase extraction; and (C) Cayman Brac sub-fossil sample number 20 following digestion with trypsin, indicating a failed result (i.e. a lack of obtainable collagen in the sample). Some peaks are labelled for interest and to demonstrate a match with *Capromys* sp.

**Fig 3:**
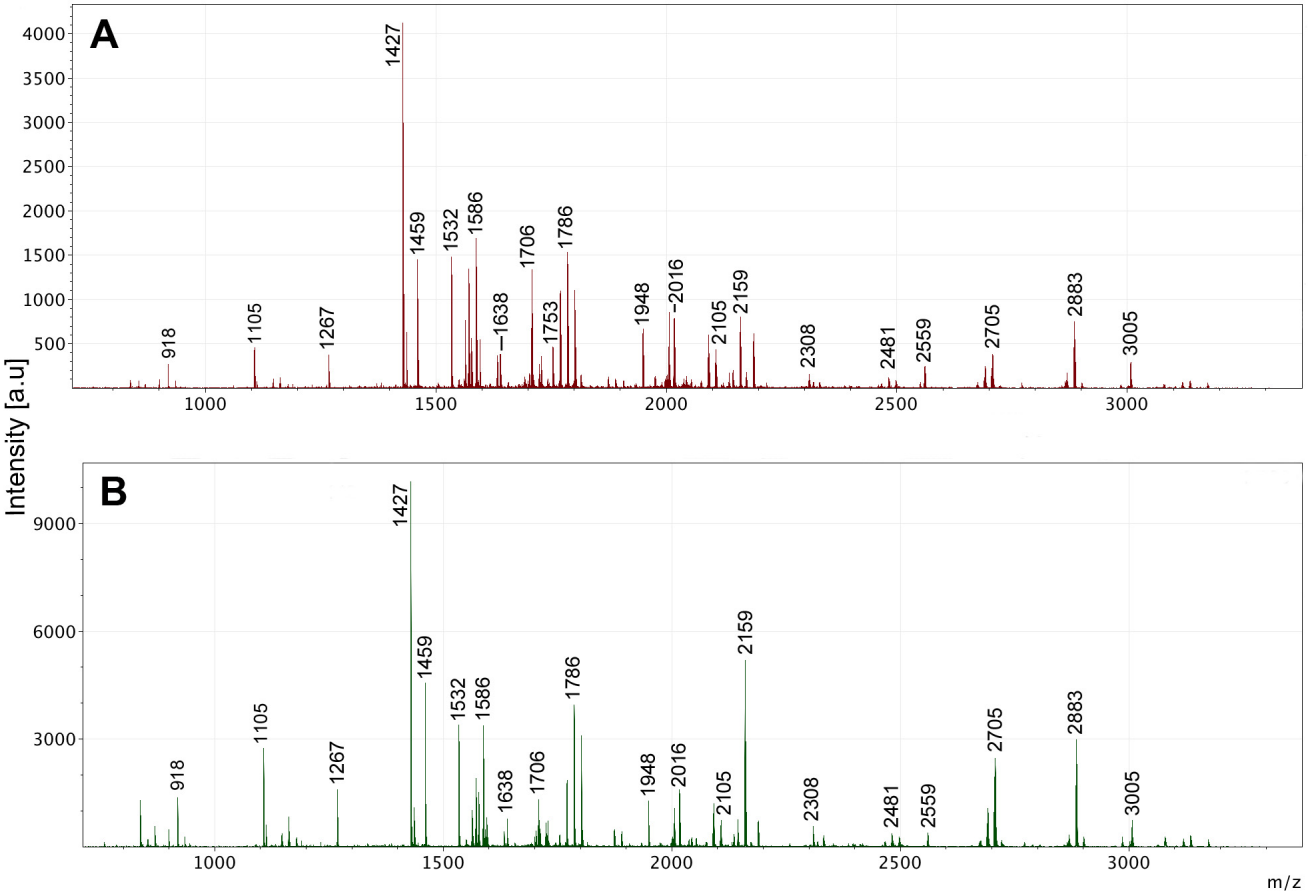
MALDI-MS spectra showing successful peptide mass fingerprints (PMFs) following 10% ACN fractionations of samples 430 (A), and 433 (B). Both samples were digested with trypsin and purified using C18 solid phase extraction. Some peaks are labelled for interest and to demonstrate a match with *Capromys* sp.

Zooarchaeological investigation of faunal remains from five caves on Cayman Brac reveals preservation of collagen in 40% (8/20) of the specimens tested (S1 Appendix); a reasonable and predictable outcome considering the prevailing tropical climate. Radiocarbon dates were successfully obtained from ancient bone material, revealing ages ranging from 393 ± 25 yr BP to 1588 ± 26 yr BP, identified as extinct hutia, *Capromys* sp., as revealed by ZooMS analysis (Table 1, Figs 2 and 3).

### ZooMS as a screening technique

ZooMS analyses of sub-fossil specimens from Cayman Brac indicate the presence of preserved collagen in bone material of >1,500 years old (Table 1, Figs 2 and 3). All of the bone samples that yielded ^14^C dates also gave excellent collagen fingerprints, and conversely those that gave poor fingerprints also failed dating. Moreover, in a second round of analysis, a batch of 81 bone fragments from various localities within Green Cave were subjected to ZooMS to pre-screen for ^14^C dating (Table 2). Of these 81 bone fragments, a total of eight produced collagen fingerprints (Table 2, Fig 3), demonstrating collagen survival in these eight specimens. Just two samples, 430 and 433, were then selected and sent to ORAU for dating. They successfully returned dates of 1134 ± 34 yr BP and 1166 ± 34 yr BP respectively.

The presence of collagen in Cayman Brac sub-fossils is indicated through the acquisition of successful PMFs (Figs 2B and 3), following trypsin digestion and soft ionisation mass spectrometry. The contrast between a successful PMF, and one that fails due to a lack of preserved collagen (Fig 2C) is clear. >10 peaks above *m/z* 1500 with S/N ratio of 5.0 (mMass) indicated a positive result. ZooMS and ^14^C dating both rely on the presence of preserved collagen in a sample to achieve a positive result, thus there is 100% matching between samples which either passed both (✓) or failed both (X) analyses. Additionally, all the bone samples analysed here show PMFs that match that of the reference sample of *Capromys pilorides*, [as shown by the common peaks between ancient and modern samples—see Fig 2A] indicating that they are likely to be remains of extinct hutia, *Capromys* sp., as yet undescribed from the Cayman Islands. It can be noted that peak height and area in modern samples is much greater than in ancient specimens, which is a common artefact of collagen degradation over time. Certain parameters are routinely tested at ORAU, including %yield, collagen C:N and %C, in order to assess a sample as worthy of dating (Table 1). Within all the Cayman Brac sub-fossil samples that were successfully dated, these parameters fell within the optimum limits for reliable dating [3].

## Discussion

### Collagen survival in the tropics

Surprisingly little work has been conducted on bone collagen preservation in the tropics, although it is well known that collagen survival is subject to a number of factors that work synergistically to facilitate degradation; namely time, temperature, bacterial presence, geochemistry and hydrology [32, 33]. Through the successful acquirement of collagen peptide mass spectra and ^14^C dates, we demonstrate that the cave systems on Cayman Brac (Cayman Islands) facilitate excellent preservation that is unusual for the tropics. ^14^C dates of up to 1588 ± 26 yr BP were obtained, which are comparable to other sites of exceptional preservation in similar equatorial climes, such as Puerto Rico [34], and other West Indian islands [35]. On the Cayman Islands, the subterranean cave systems may inhibit biomaterial breakdown pathways, allowing a higher degree of preservation than would be expected from terrestrial locations in the tropics.

Radiocarbon dating can be a particularly costly pursuit in regions whereby collagen survival is limited and poorly understood. ZooMS provides an alternative rapid screening technique that can be utilised to identify collagen integrity (see Fig 2) and can be used as a sample selection procedure prior to ^14^C dating. The method has so far exhibited a 100% success rate with regards to successfully categorising samples as suitable for dating or not, a figure significantly larger than the success rates achieved via %N or C:N investigations [11]. ZooMS can reduce time and expense, whilst lowering the risk of unreliable date attainment, preventing unnecessary sample destruction and providing additional information on species identification. The time required for date acquisition from ^14^C laboratories may be many months but can be saved with ZooMS analysis prior to dating, reducing the pressure of sample pre-screening on the laboratory. As both ^14^C and ZooMS techniques rely on collagen preservation for accurate results, a successful result in one technique is likely to produce a successful result in the other. PMF quality (peak number and height) is largely reflected by the quantity of preserved collagen, and a sample of <1% collagen yield that would be rejected for dating at ORAU for example, could still yield a collagen fingerprint, albeit very poor. The application of a signal-to-noise (S/N) filter and a threshold of 10+ peaks required above *m/z* 1500 during data analysis screens out samples that produce poor PMFs and would be more likely to fail radiocarbon dating.

Fossil samples from Green Cave show a trend for increasing specimen age with increasing distance from the cave entrance (see Table 1). In the first round of dating (sample numbers 3 to 25), samples were selected visually based upon the extent of mineralisation (e.g. Fig 1) and were only then subjected to ZooMS analyses following ^14^C results. Within this first round, four samples (numbers 10 to 15) were tested from Green Cave, Chamber 5, all of which failed dating and ZooMS. In the second round of dating, samples 430 and 433 were selected from a suite of 81 bone fragments all from Green Cave, Chamber 5. Samples 430 and 433 both passed ZooMS analyses and went on to be successfully dated at ORAU (Table 1). Therefore, despite the lack of ^14^C results from Chamber 5 in the first round of dating (where more samples were selected for analysis than for any other chamber), we demonstrate with samples 430 and 433 that ZooMS can be successfully utilised as a pre-screening technique to highlight samples likely to yield ^14^C dates.

It is noteworthy that samples collected from Chamber 4 and Chamber 5 of Green Cave are older than those from chambers nearer the cave entrance. This trend is not exclusive, as the two Chamber 5 samples, 430 and 433, are moderately younger (by approximately 400 years) than the successfully dated Chamber 4 specimen, sample 7. This is not a surprising finding due to the high levels of bioturbation within the cave systems of Cayman Brac. A fissure is also present at the furthest end of Chamber 5 and these younger dates may be when the fissure opened, allowing animals a new access to the cave. Older samples are likely to be more taphonomically altered, perhaps being deposited during a period when mineral encapsulation was not equally occurring, increasing the value of ZooMS in highlighting sample suitability for dating when faced with a large assemblage of bone fragments of unknown preservation status. Fresh bone naturally yields a collagen content (%yield) that is approximately 22% the total weight of the bone [3]. The samples in this study that passed the %yield test (i.e. presented a figure of >1% wt) had an average of 7.66% wt across the eight samples, indicating a good level of preservation considering the depositional environment. Sample number 7, the oldest sample, (Fig 2B), produced a date of 1588 ± 26 yr BP and a %yield of 1.3% wt (i.e. just above the minimum threshold) which suggests a rough guideline to the maximum age a Cayman Brac sub-fossil sample may reach before collagen becomes taphonomically altered to a degree that it will fail ZooMS and ^14^C dating. It is also significant that %C and collagen C:N data for the eight samples are in keeping with thresholds that demonstrate dates are reliably yielded from endogenous collagen [3]. Steadman and Morgan [25] dated stratigraphic layers down to 160 cm in Patton’s Fissure (north Cayman Brac) up to 13,850 ± 135 yr BP. Though this layer is much deeper than those collected for this study (i.e. context 4, down to 35 cm), it suggests that ZooMS can be used to hone in on exceptionally preserved remains that are suitable for dating, of potentially greater age than our current study. Such future work could help solidify the understanding of: (i) the temporal and spatial range of collagen survival in the tropics, (ii) last occurrence dates for key extinct taxa on Cayman Brac, and (iii) human-arrival extinction events on the Cayman Islands.

### Palaeobiodiversity through ^14^C and ZooMS analysis

It is noteworthy that all previous investigations on the faunal assemblages of Cayman Brac have relied exclusively on the presence of key morphological characteristics to undergo species identification, placing obvious constraints on the identification of remains that have become fragmented and/or heavily encased in matrix so that they have no morphologically identifiable regions visible. ZooMS is therefore uniquely suited to identifying taxa based upon fragmented samples such as those present in the cave systems of Cayman Brac.

Through the application of ZooMS, we are able to rapidly determine faunal identity and collagen integrity within bone remains, the latter for the acquirement of a temporal framework. With such insight, we have the opportunity to investigate palaeobiodiversity through time on the Cayman Islands in order to understand and offer protection to the subterranean cave ecosystem on Cayman Brac. The data derived from this and future studies will be of paramount importance in better understanding anthropogenically-driven terrestrial extinctions and last occurrence dates. It is our hope that the results from this study will demonstrate the importance of the Cayman Island cave deposits, but more importantly inform decision makers who might generate legislation to protect these fragile cave systems within this globally important biodiversity hotspot.

## Conclusions

Zooarchaeology offers a unique perspective to understanding how biodiversity has changed in response to key parameters, such as geological time and prevailing geochemistry, along with climatic or anthropogenic fluctuations through time. Type I collagen is one of the hardiest proteins, and from it we can deduce species identification (particularly where morphological recognition is no longer viable due to similar skeletal elements from other taxa present, or fragmentation), age at deposition, and a suite of other informative applications. Such insight permits identification of taxa that have become introduced, extinct or extirpated within a temporal framework. This understanding can provide supporting evidence for reintroductions or culls, towards the regeneration of natural biotas. With such knowledge, we can also improve our understanding of anthropogenic impacts on Cayman Brac and to other comparable biomes, and be better positioned to identify and protect biodiversity at these key hotspots. More significantly, the screening technique has the potential to be of worldwide significance for building chronological frameworks in the late Pleistocene.

## Supporting Information

S1 Appendix MALDI peptide mass fingerprints of the collagen extracted from radiocarbon-dated specimens following digestion with trypsin. (DOCX)
