# Supplementary material for: Collagen Fingerprinting: A New Screening Technique for Radiocarbon Dating Ancient Bone
Source: PLoS One. 2016 Mar 3;11(3):e0150650. doi: 10.1371/journal.pone.0150650 (PMC4777535; doi:10.1371/journal.pone.0150650)
Supplement: S1 Appendix — (DOCX) [file pone.0150650.s001.docx]

**S1 Appendix – MALDI peptide mass fingerprints of the collagen extracted from radiocarbon-dated specimens following digestion with trypsin.**

| #3  10% | 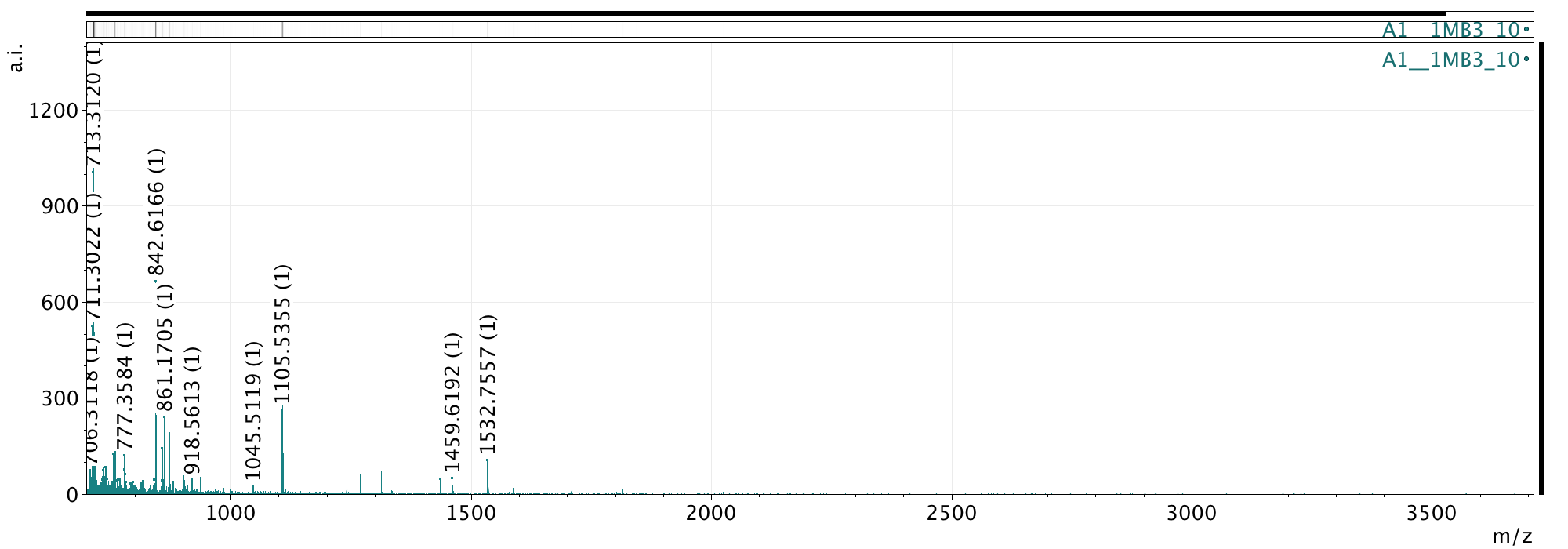 |
| --- | --- |
| #3  50% | 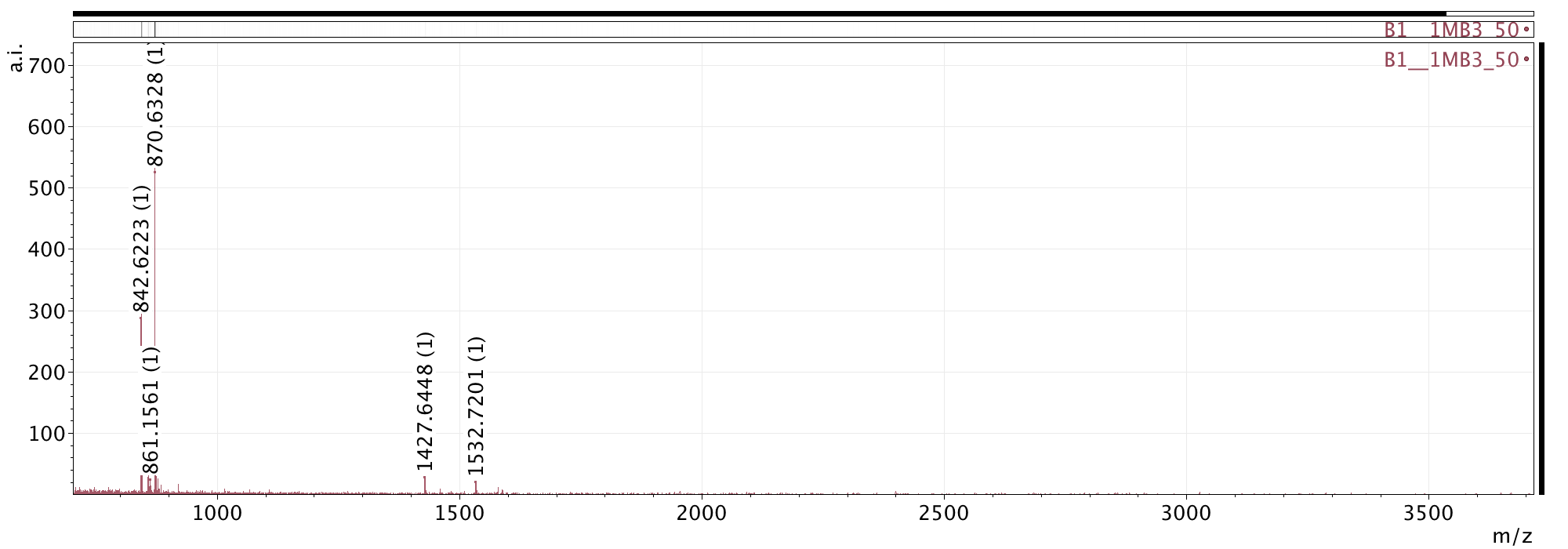 |
| #4  10% | 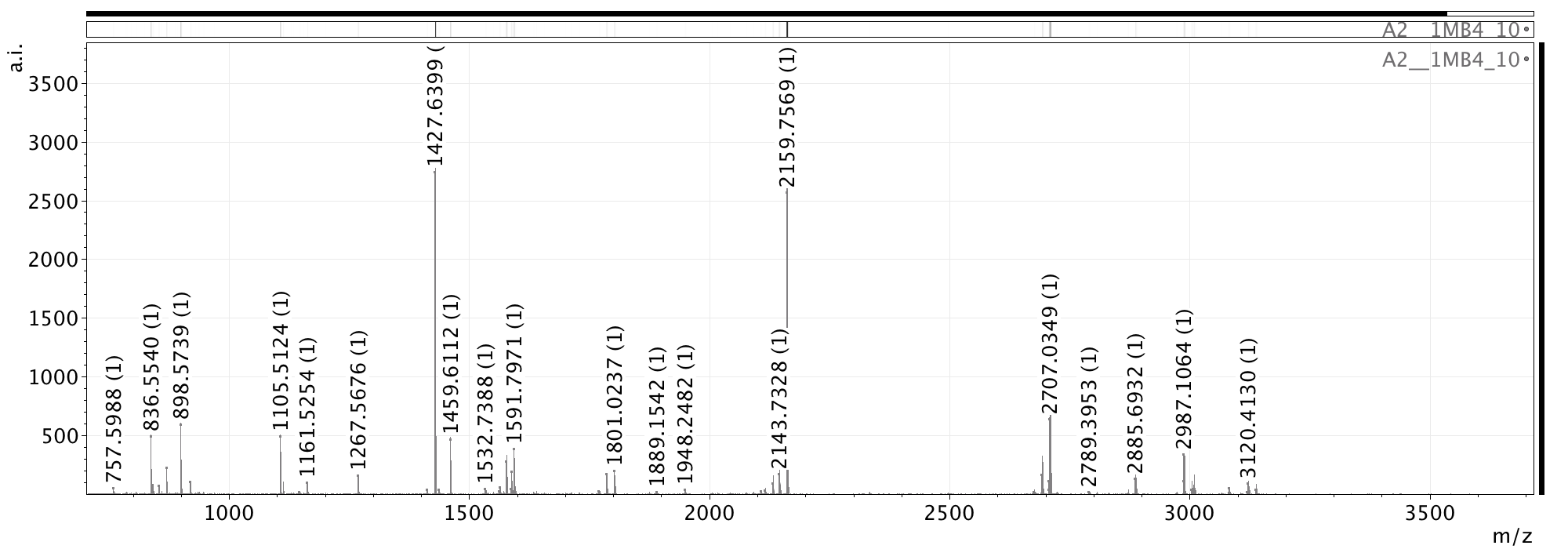 |
| #4  50% | 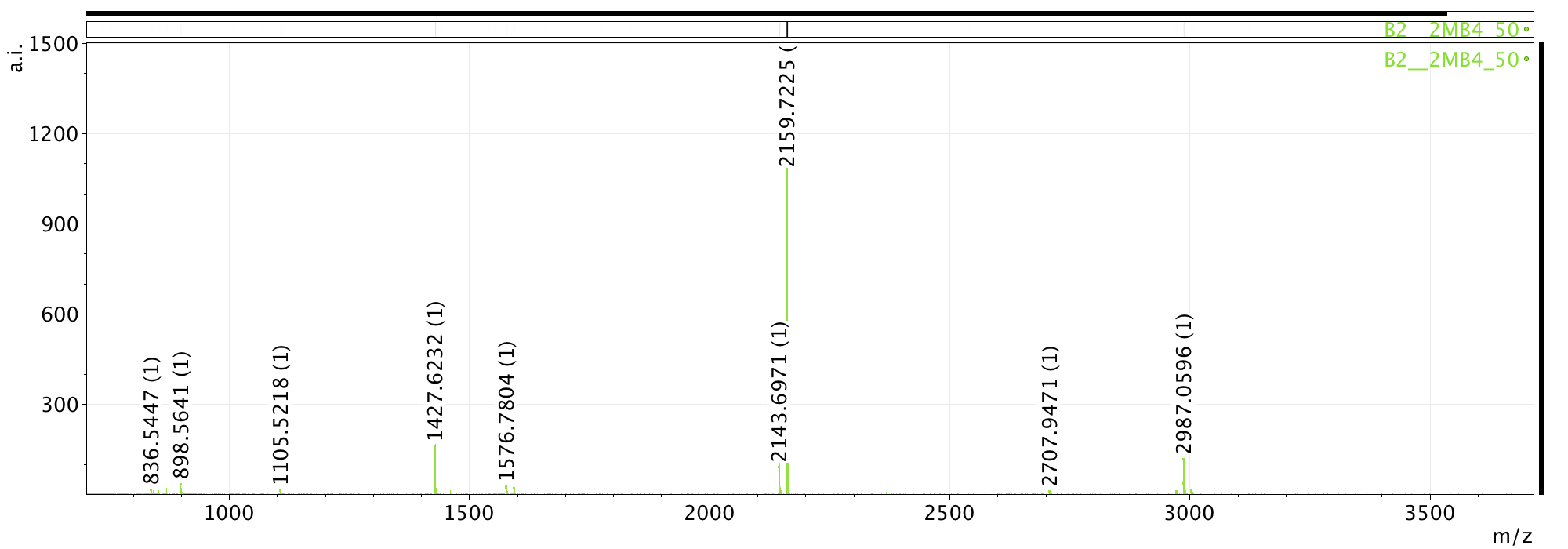 |
| #5  10% | 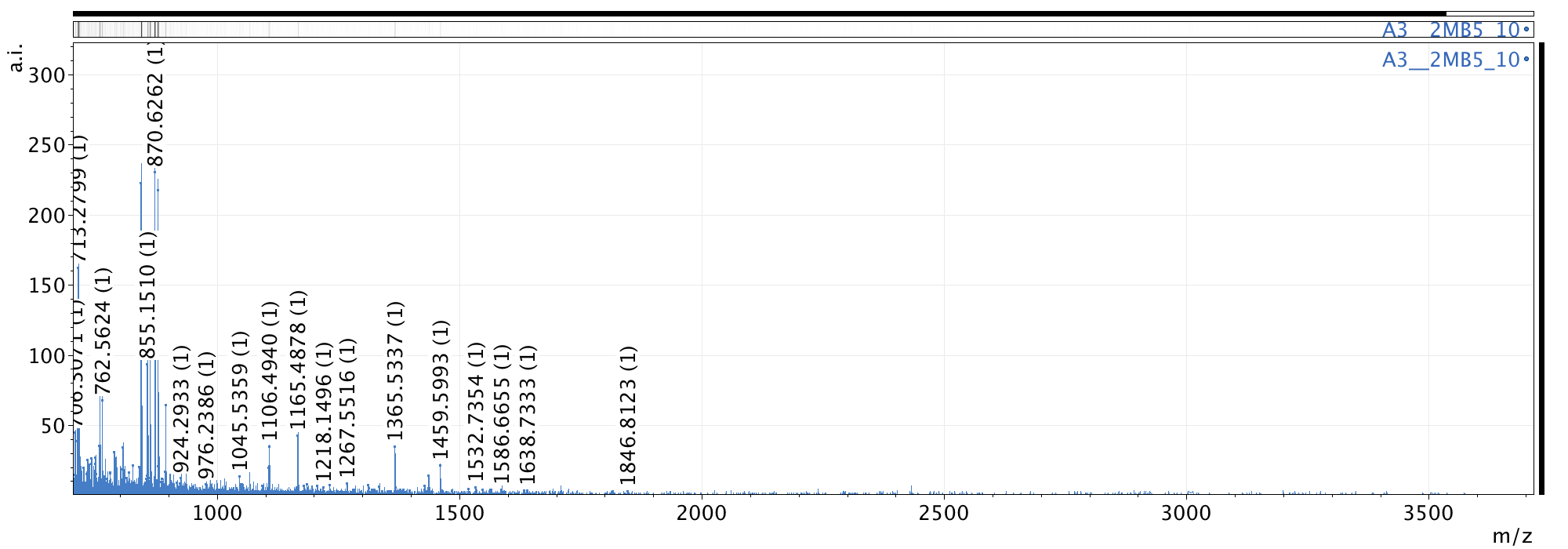 |
| #5  50% | 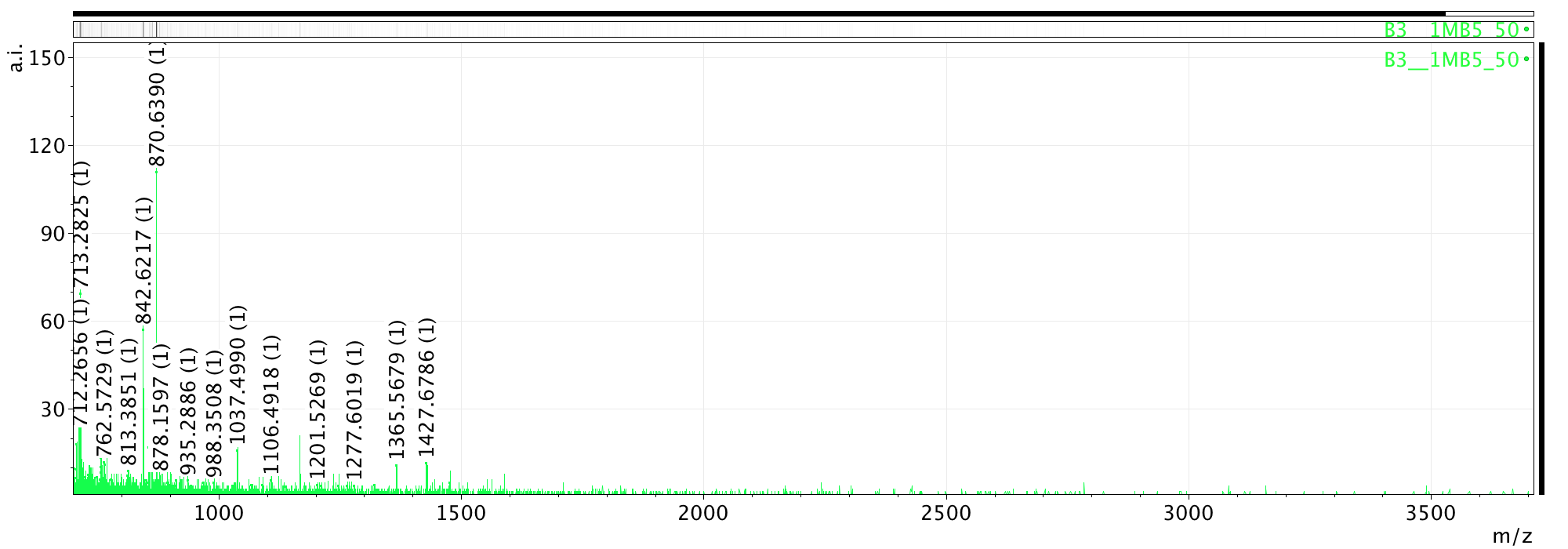 |
| #6  10% | 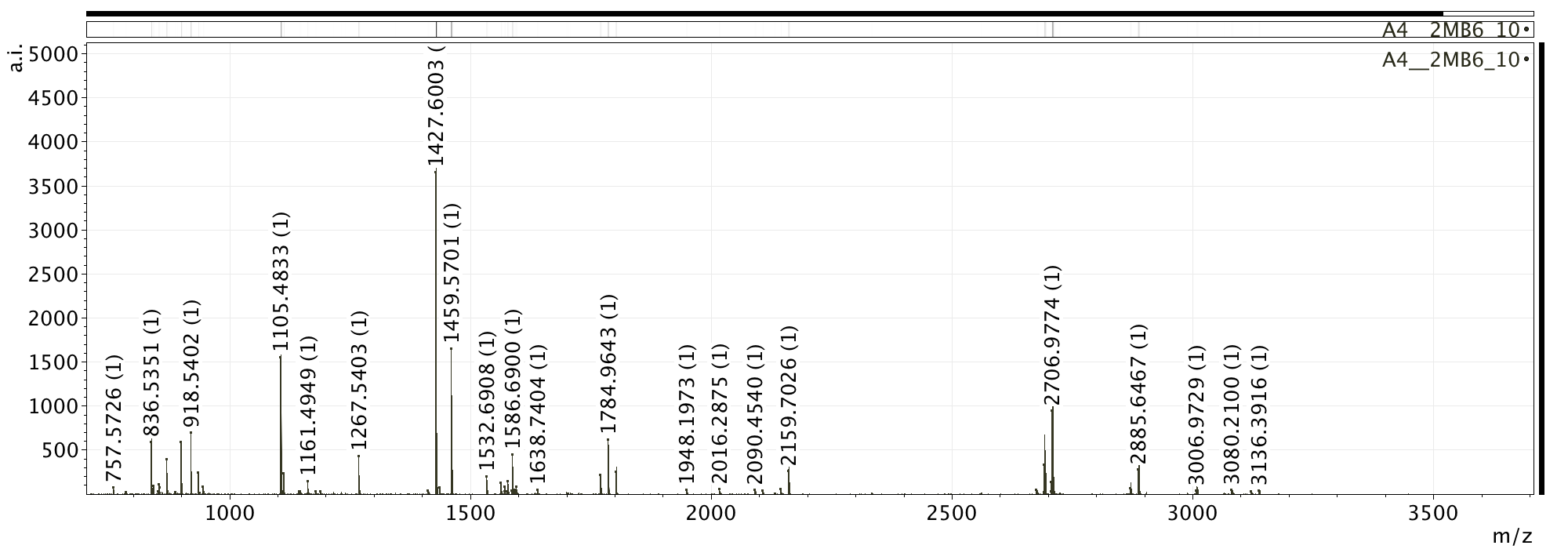 |
| #6  50% | 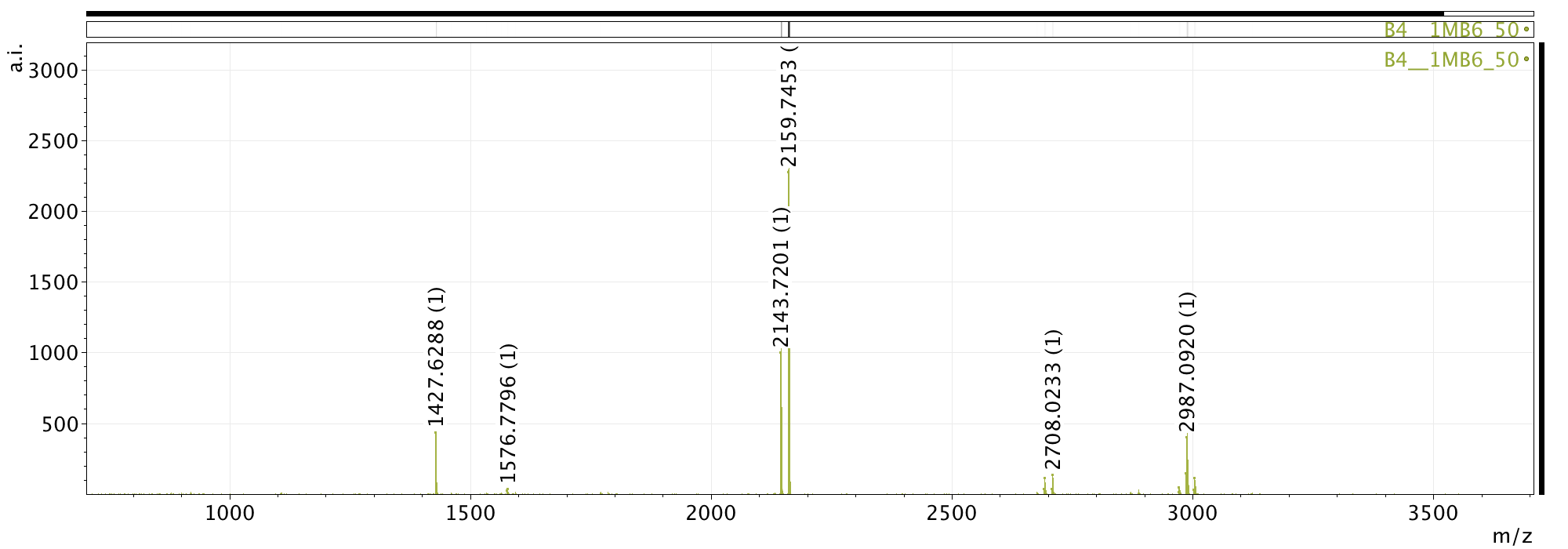 |
| #7  10% | 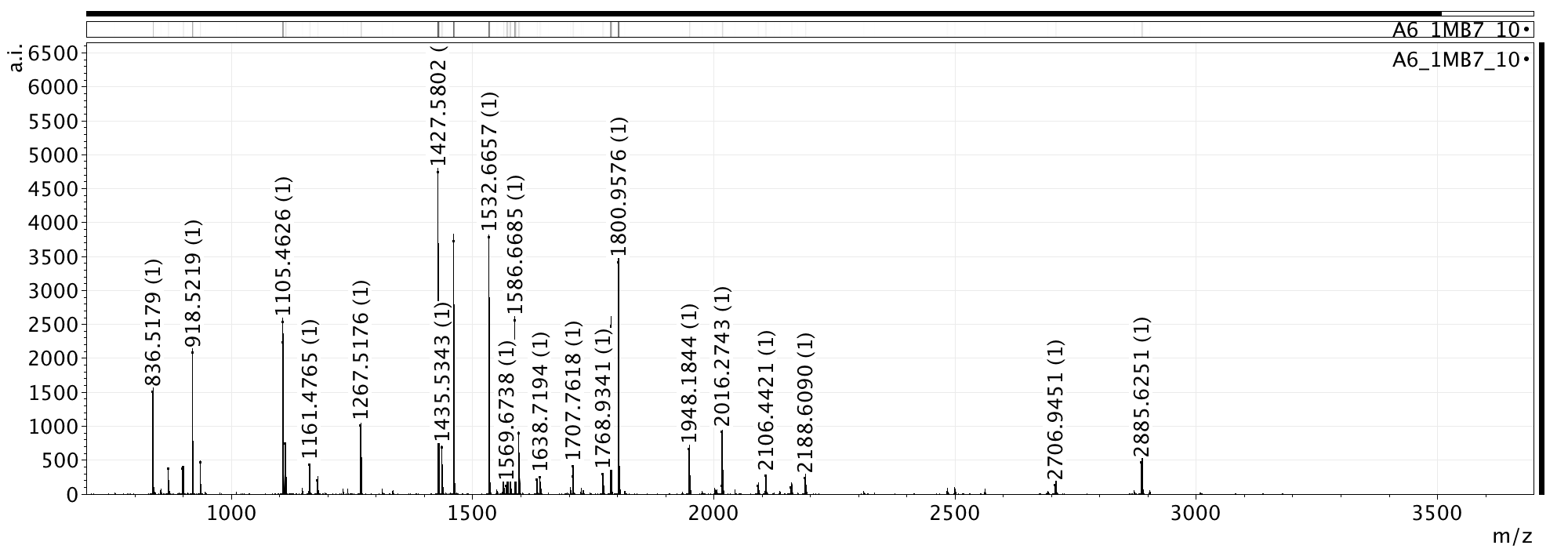 |
| #7  50% | 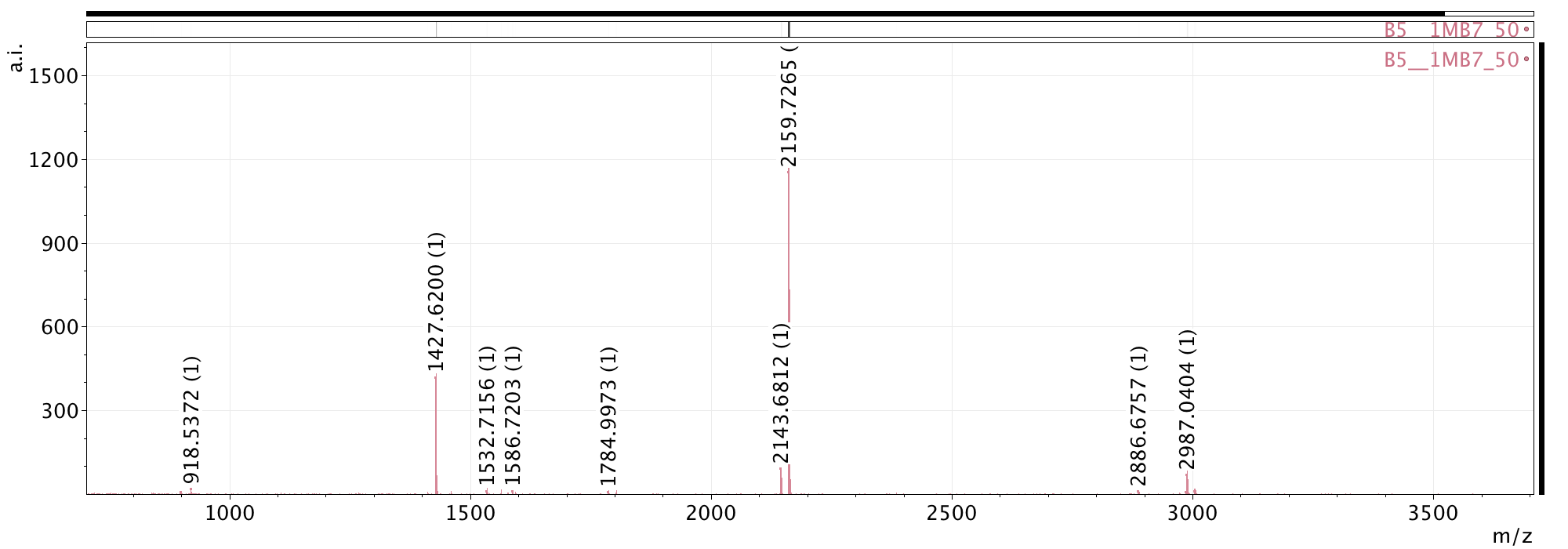 |
| #8  10% | 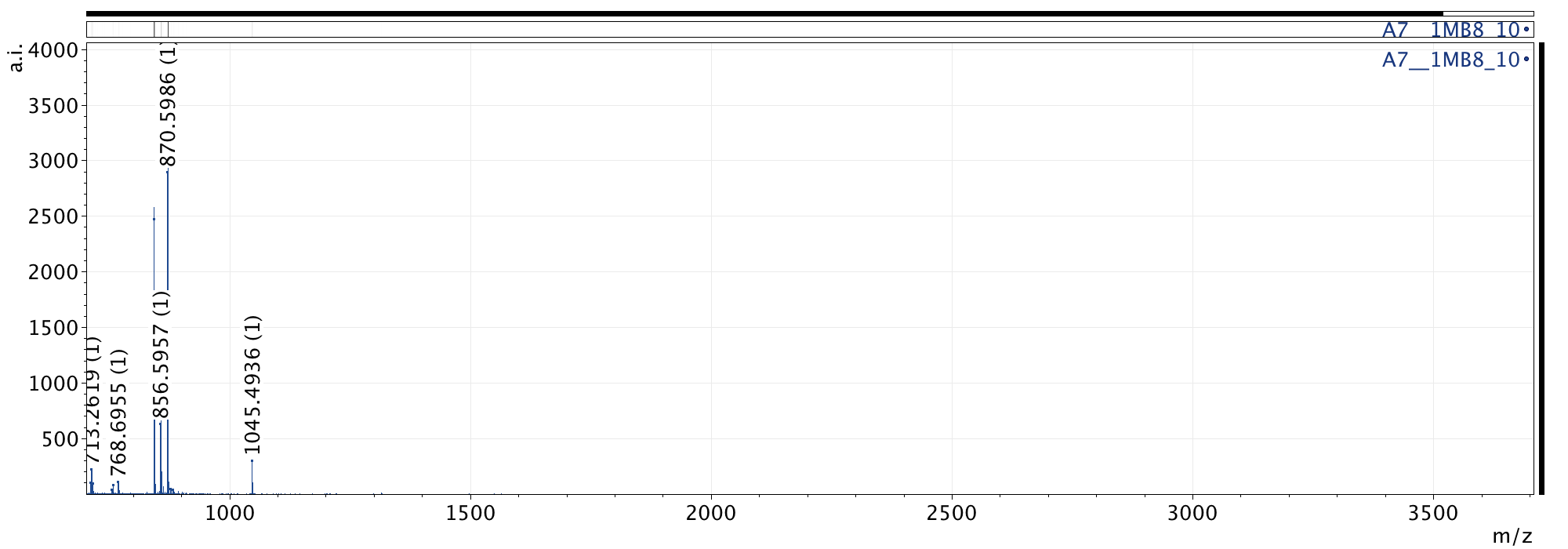 |
| #8  50% | 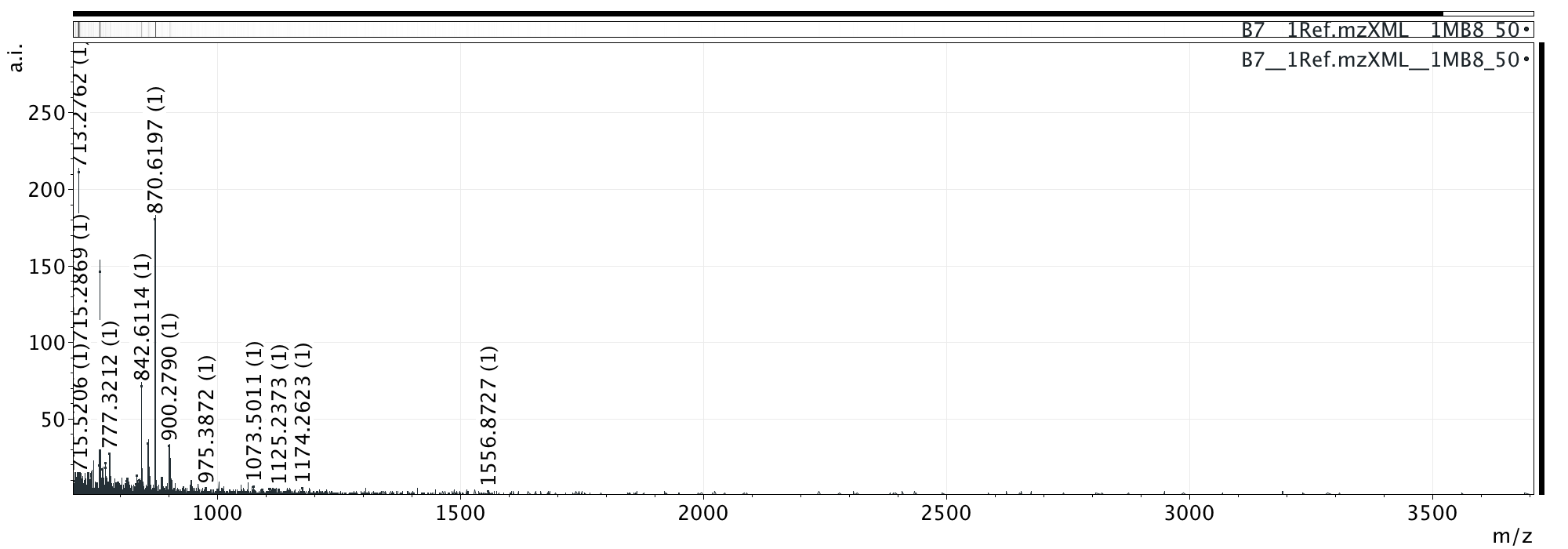 |
| #10  10% | 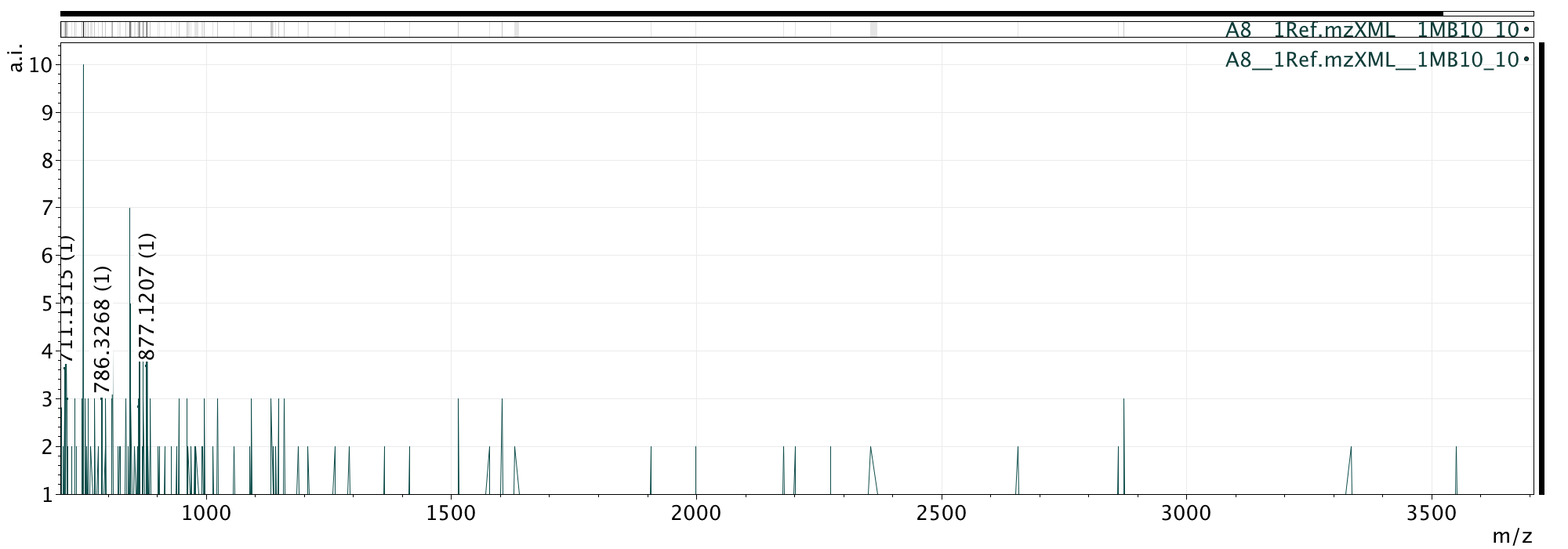 |
| #10  50% | 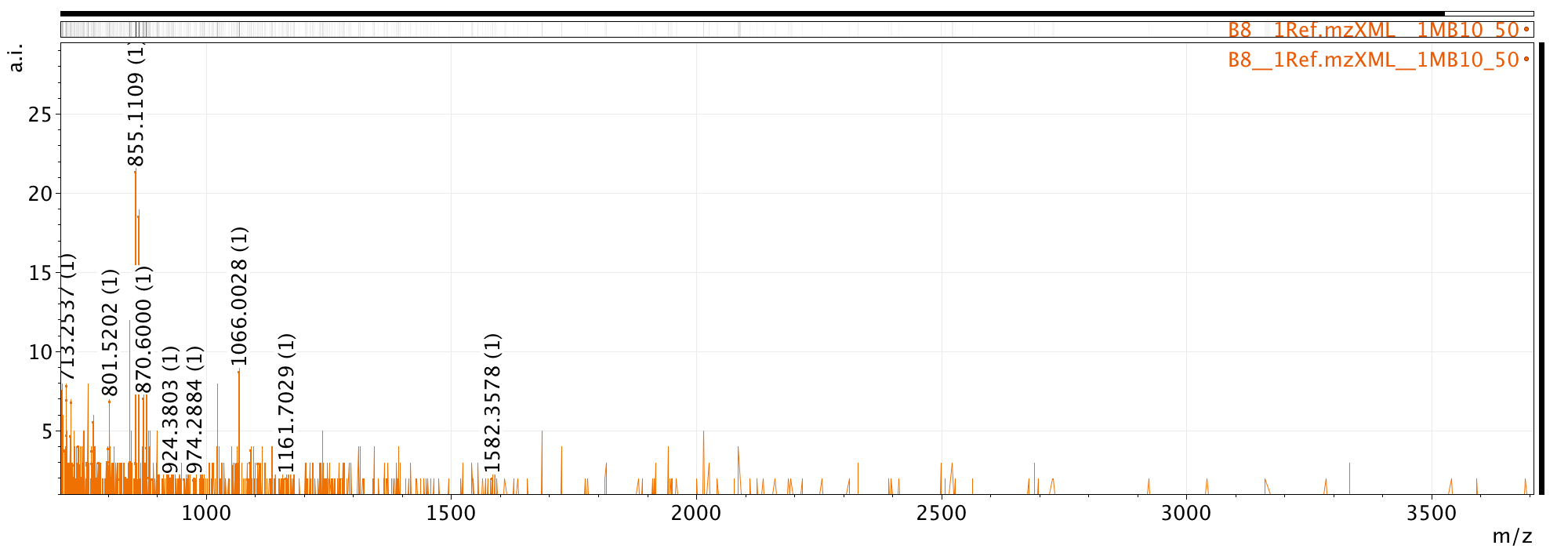 |
| #11  10% | 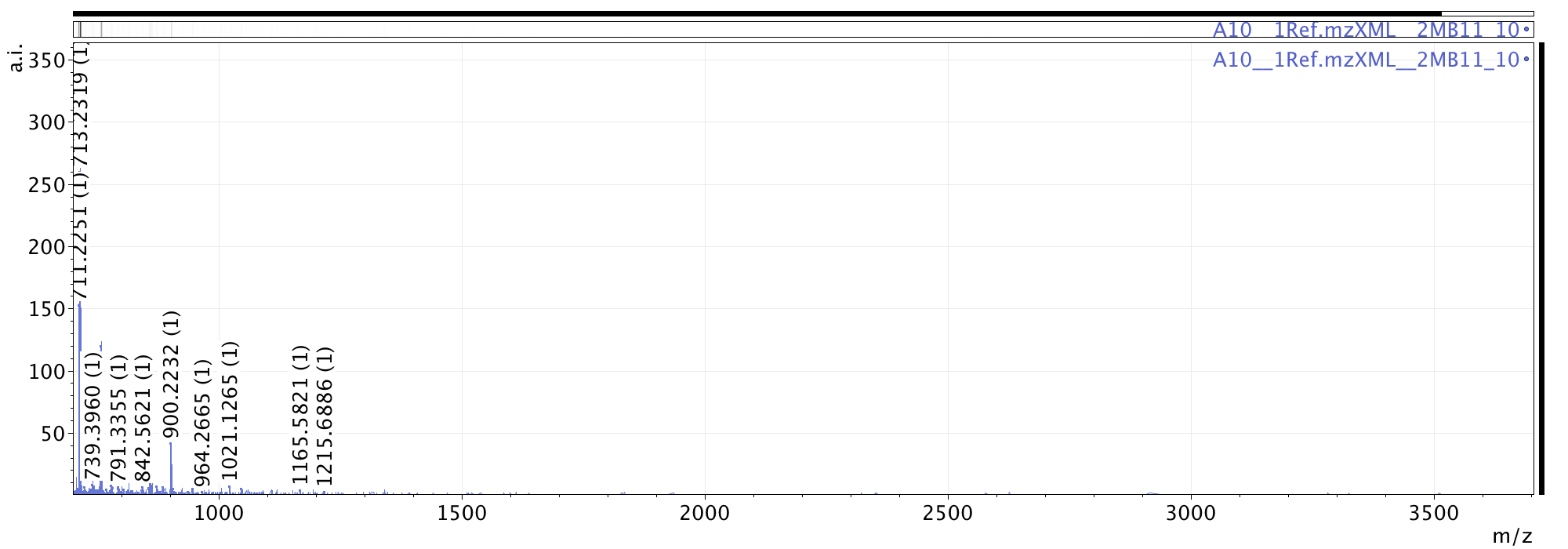 |
| #11  50% | 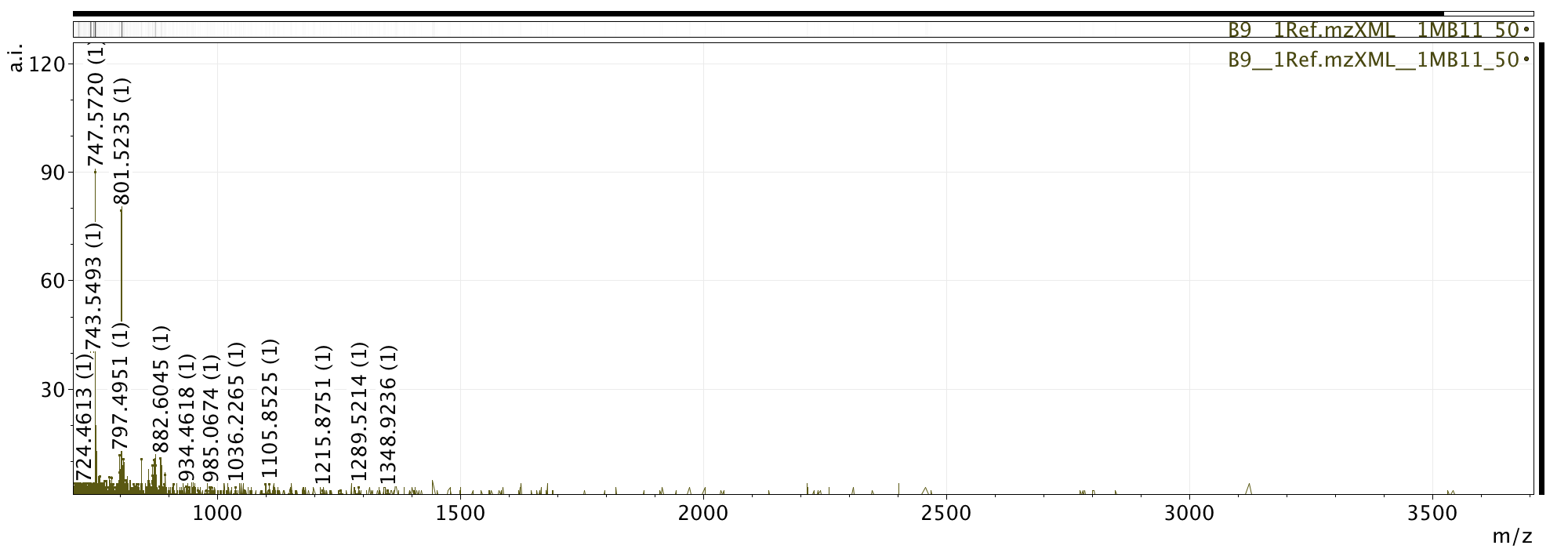 |
| #14  10% | 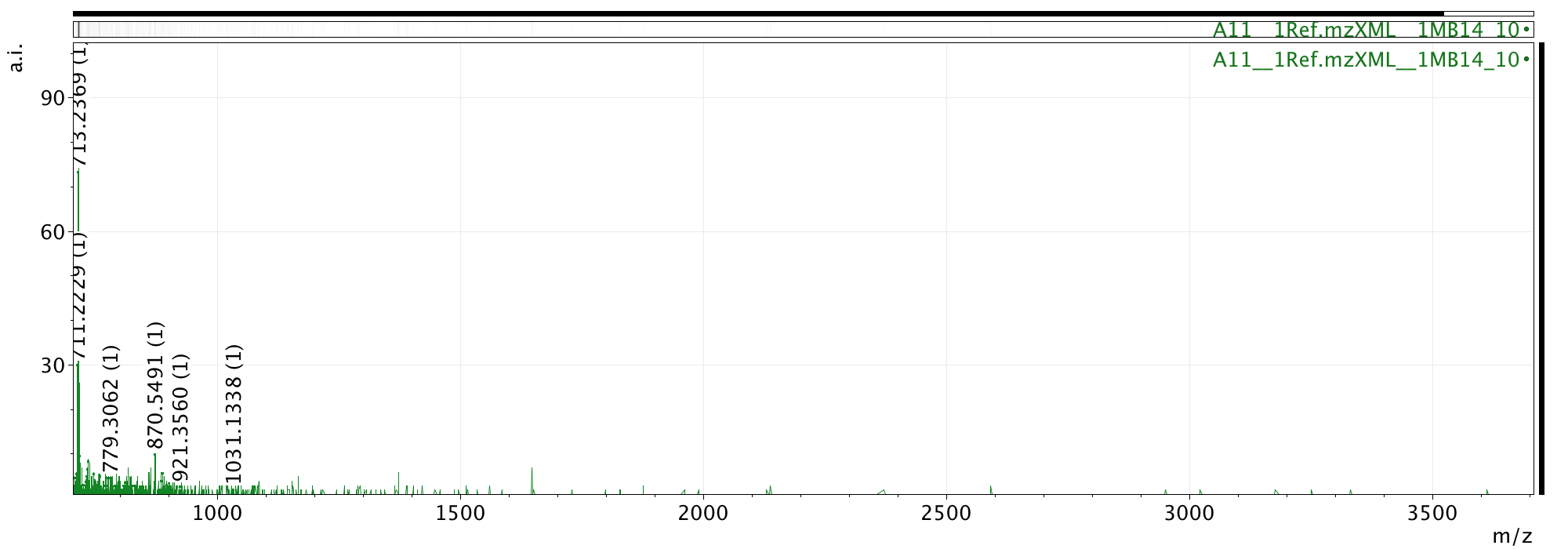 |
| #14  50% | 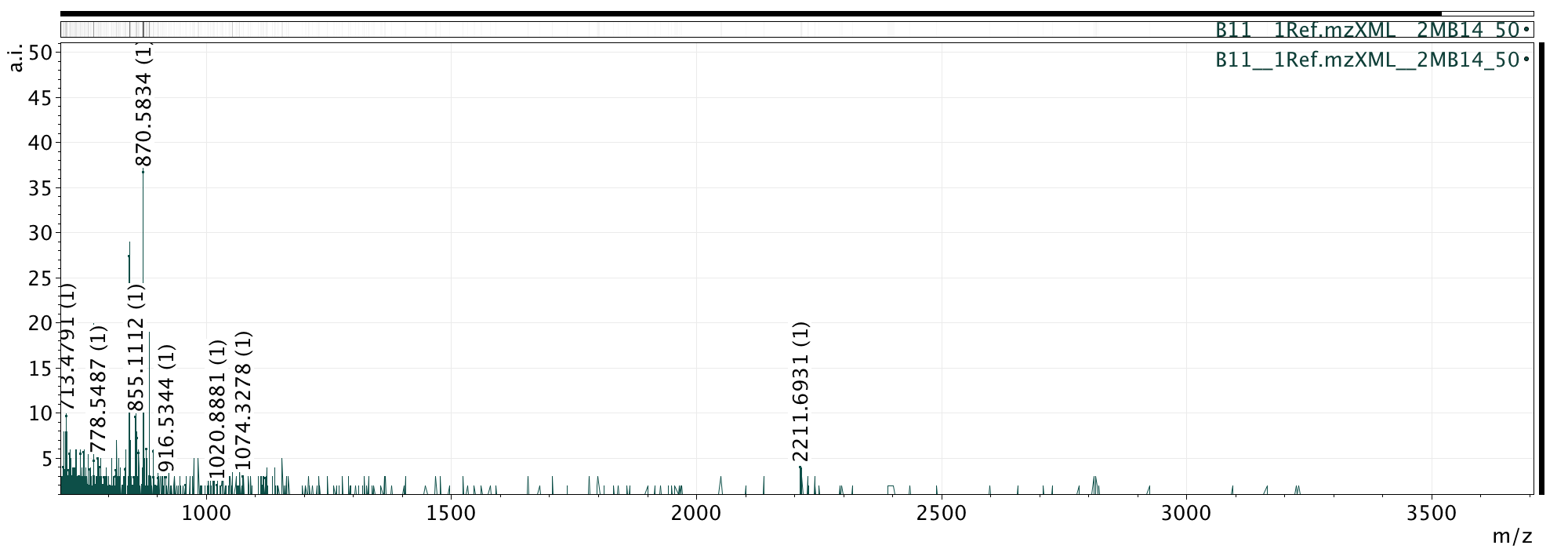 |
| #15  10% | 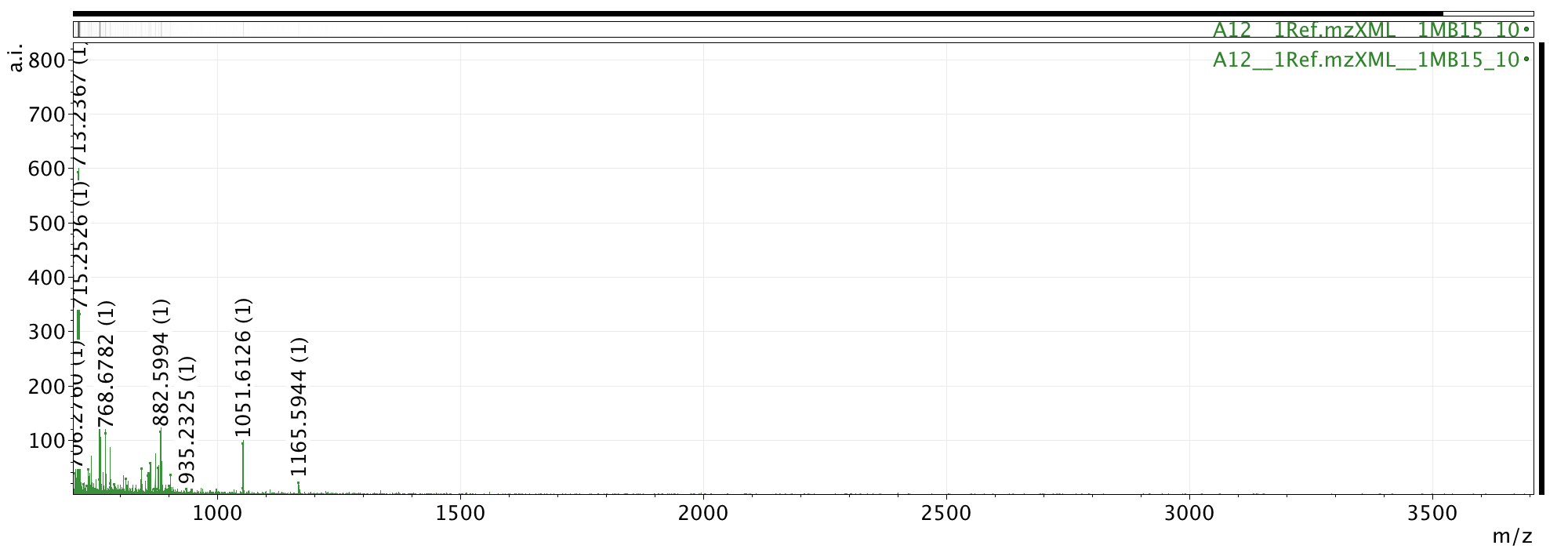 |
| #15  50% | 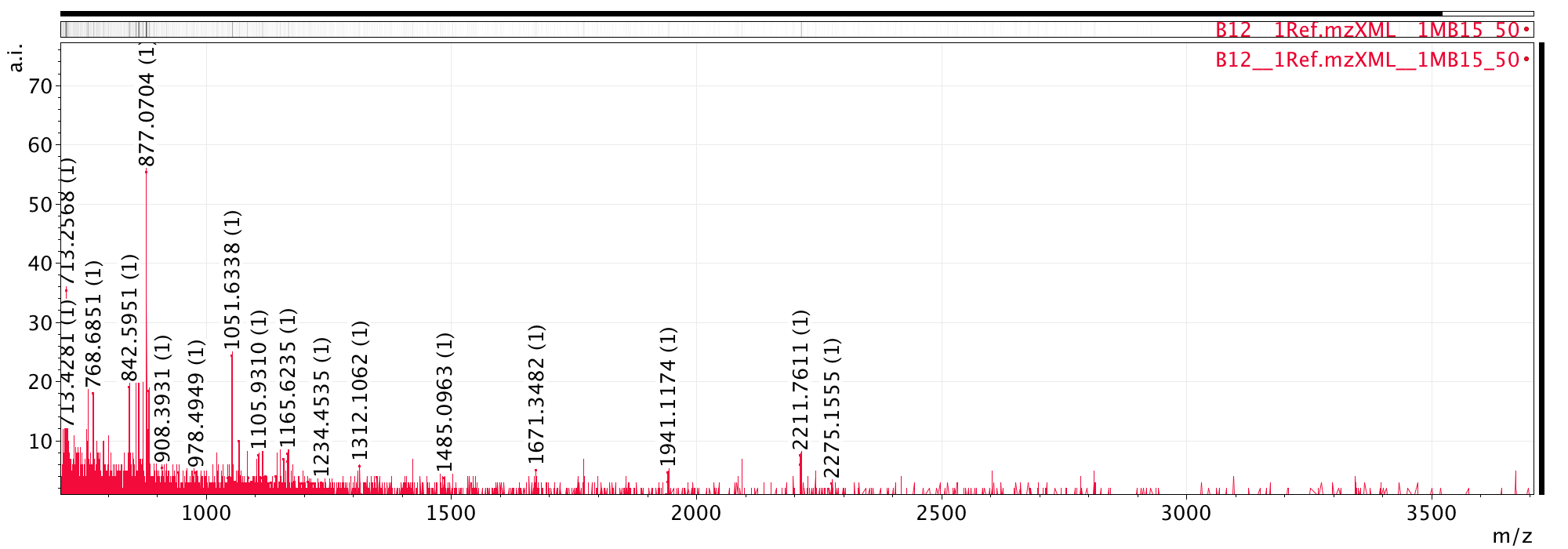 |
| #17  10% | 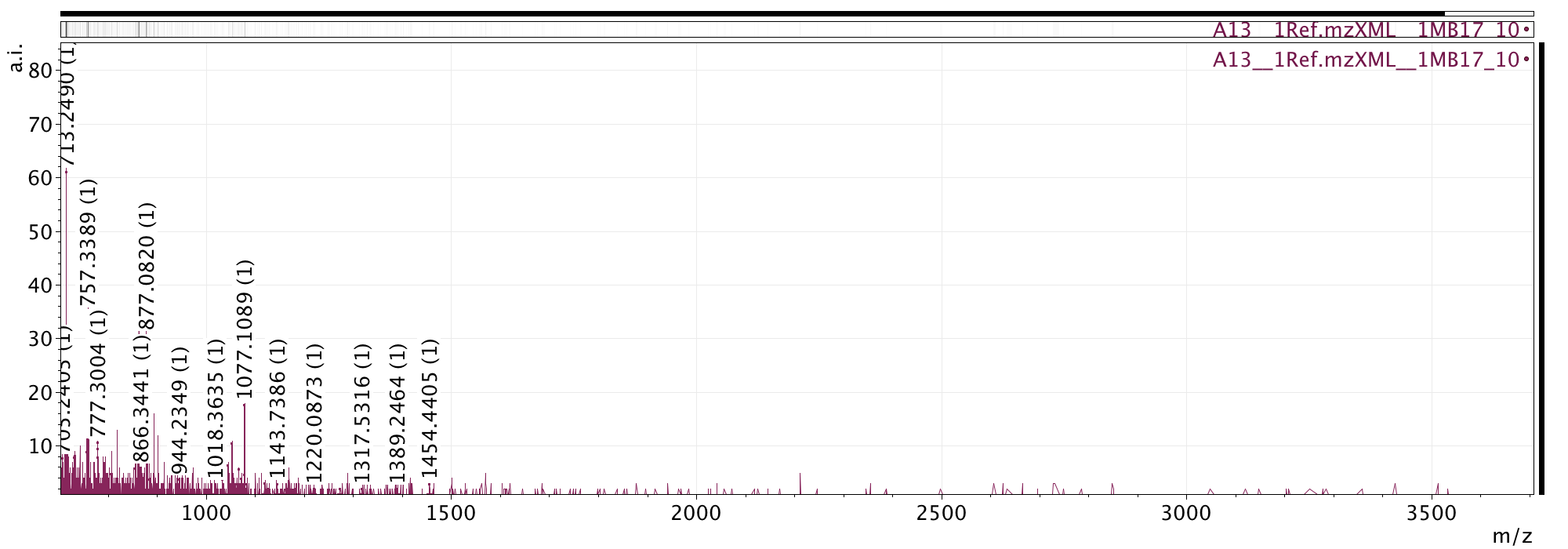 |
| #17  50% | 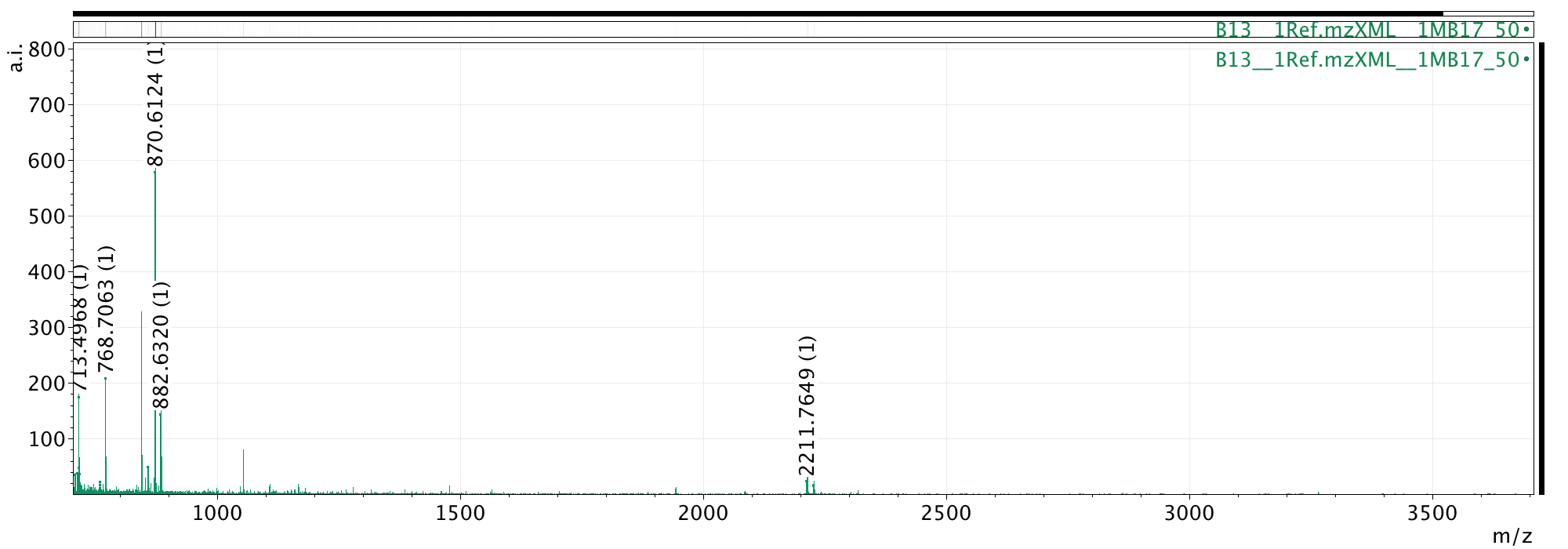 |
| #18  10% | 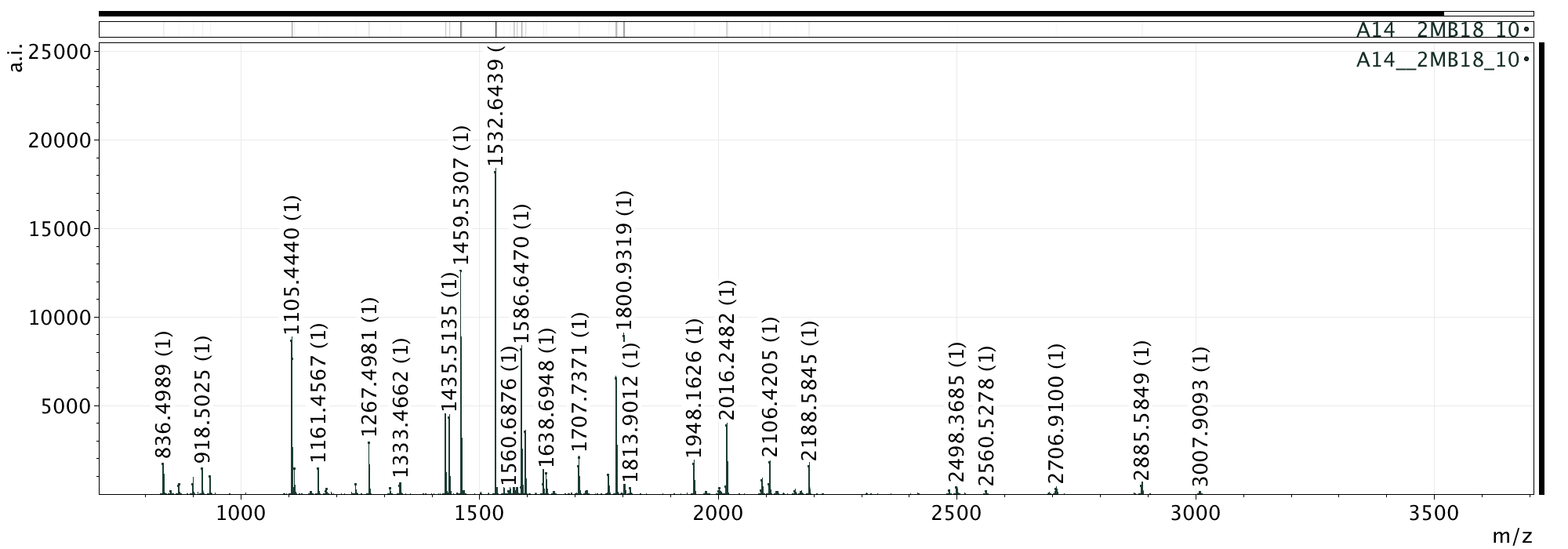 |
| #18  50% | 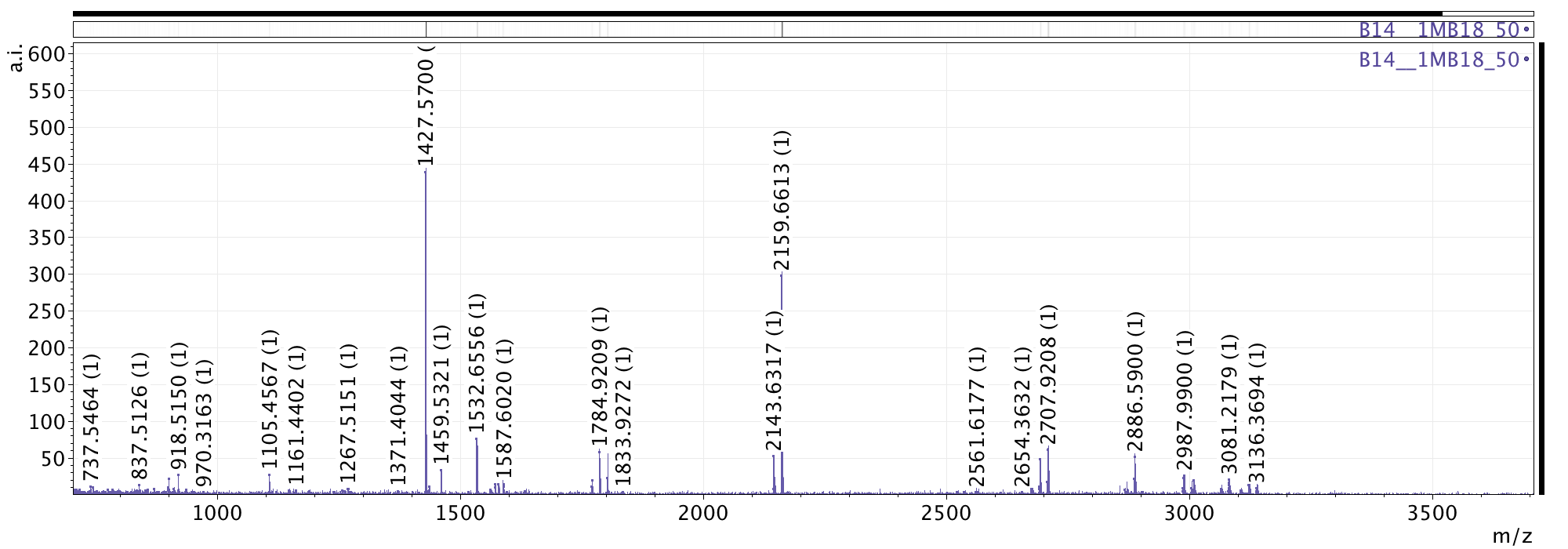 |
| #19  10% | 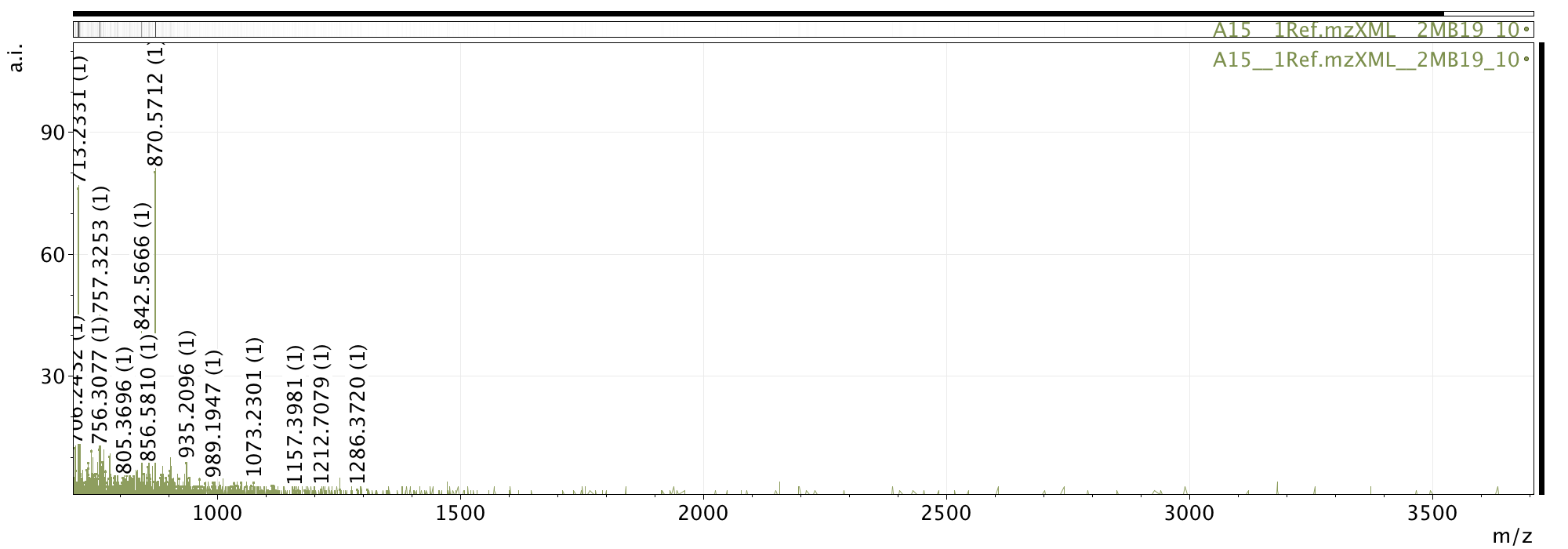 |
| #19  50% | 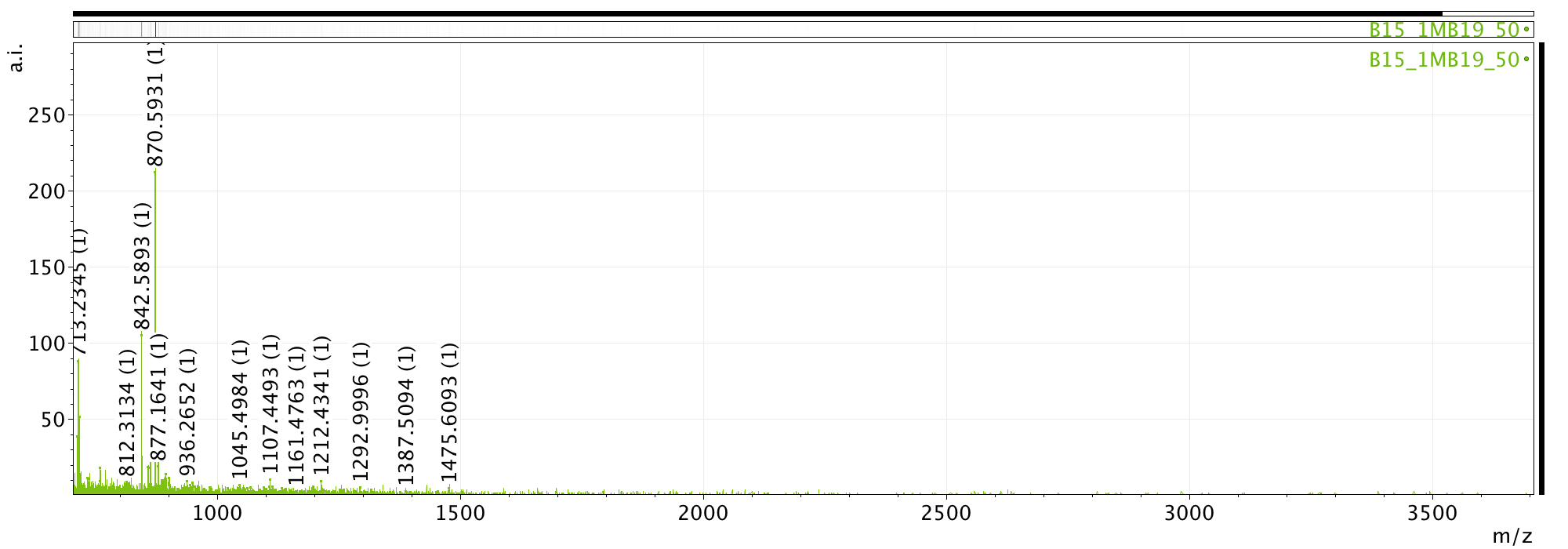 |
| #20  10% | 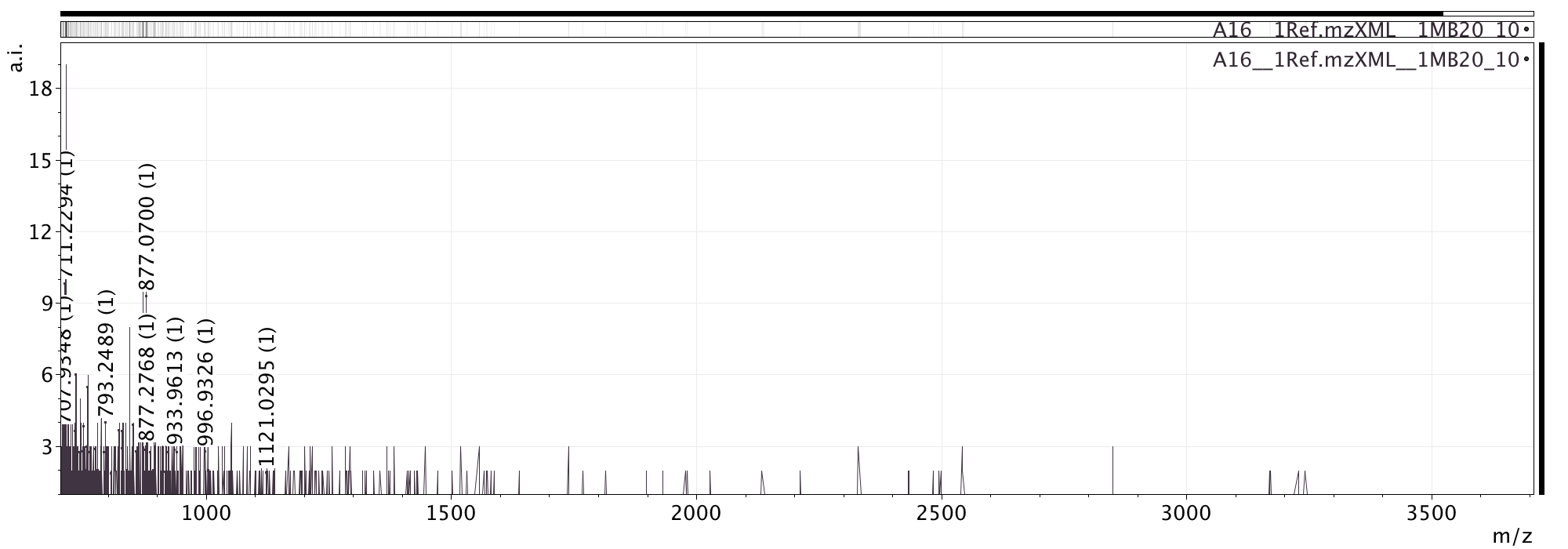 |
| #20  50% | 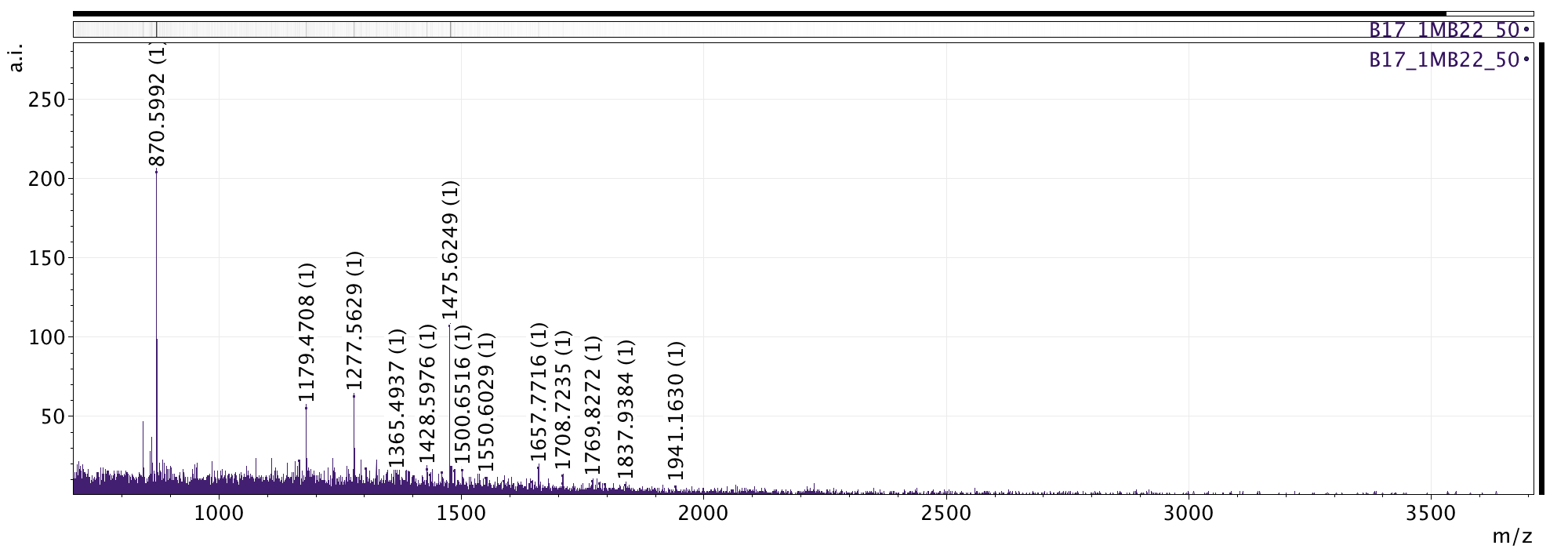 |
| #22  10% | 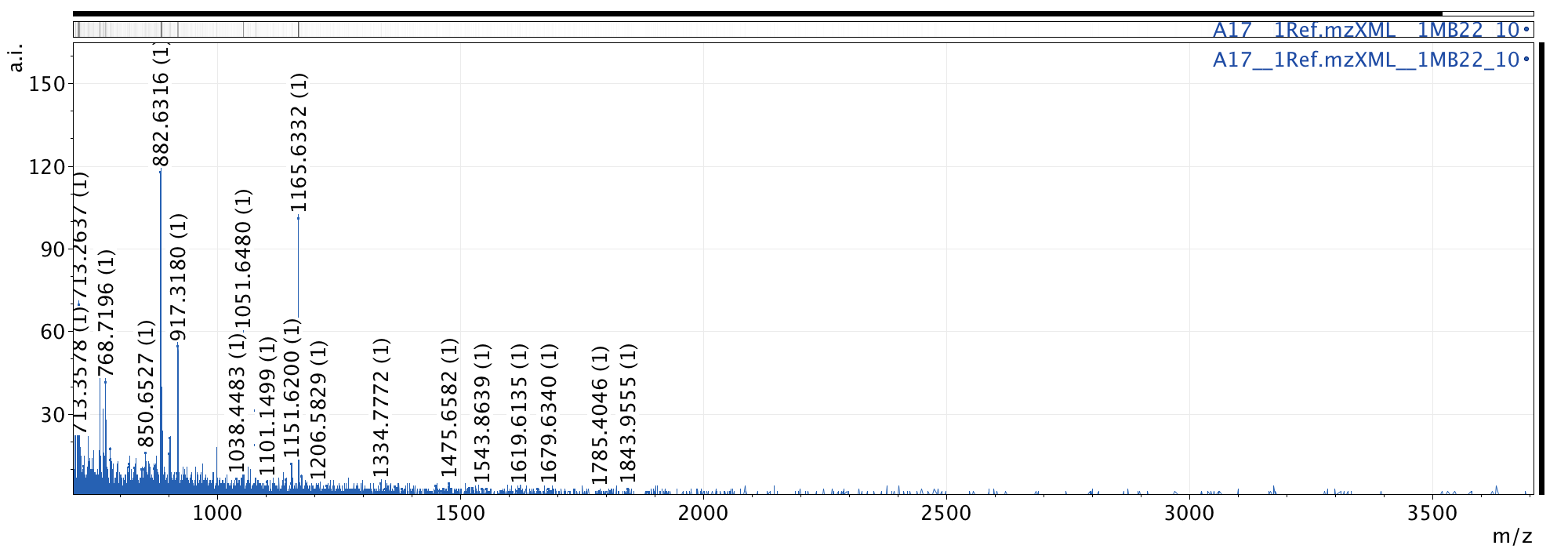 |
| #22  50% | 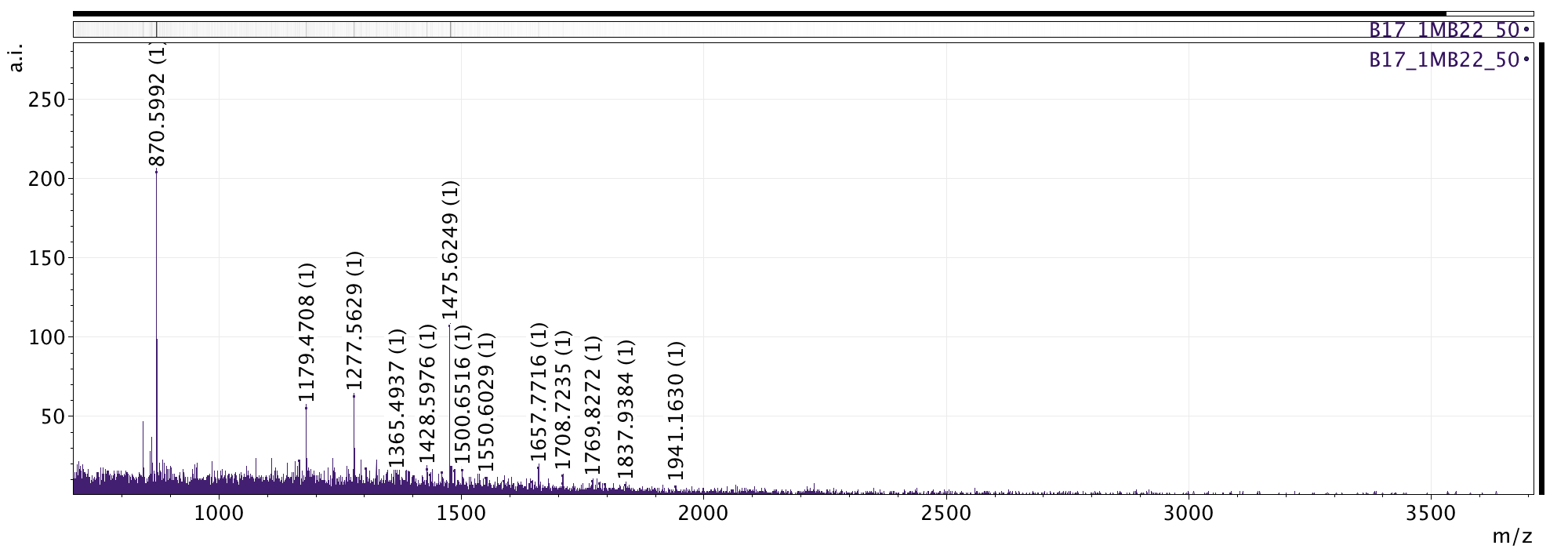 |
| #23  10% | 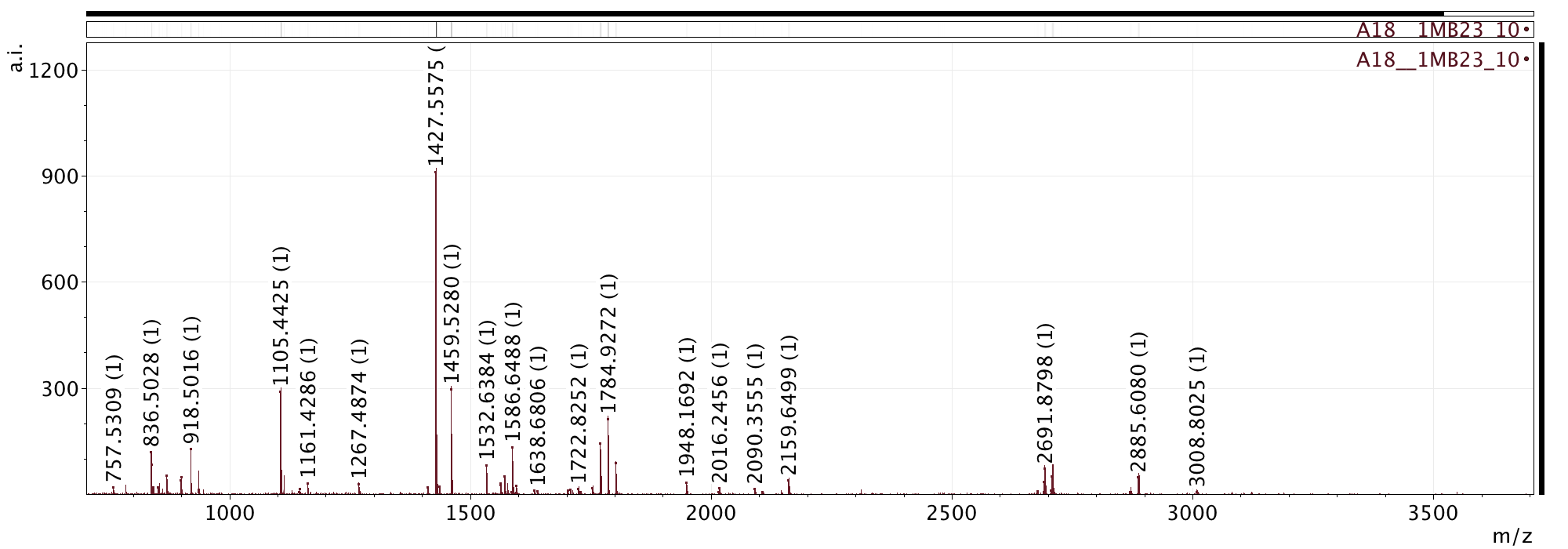 |
| #23  50% | 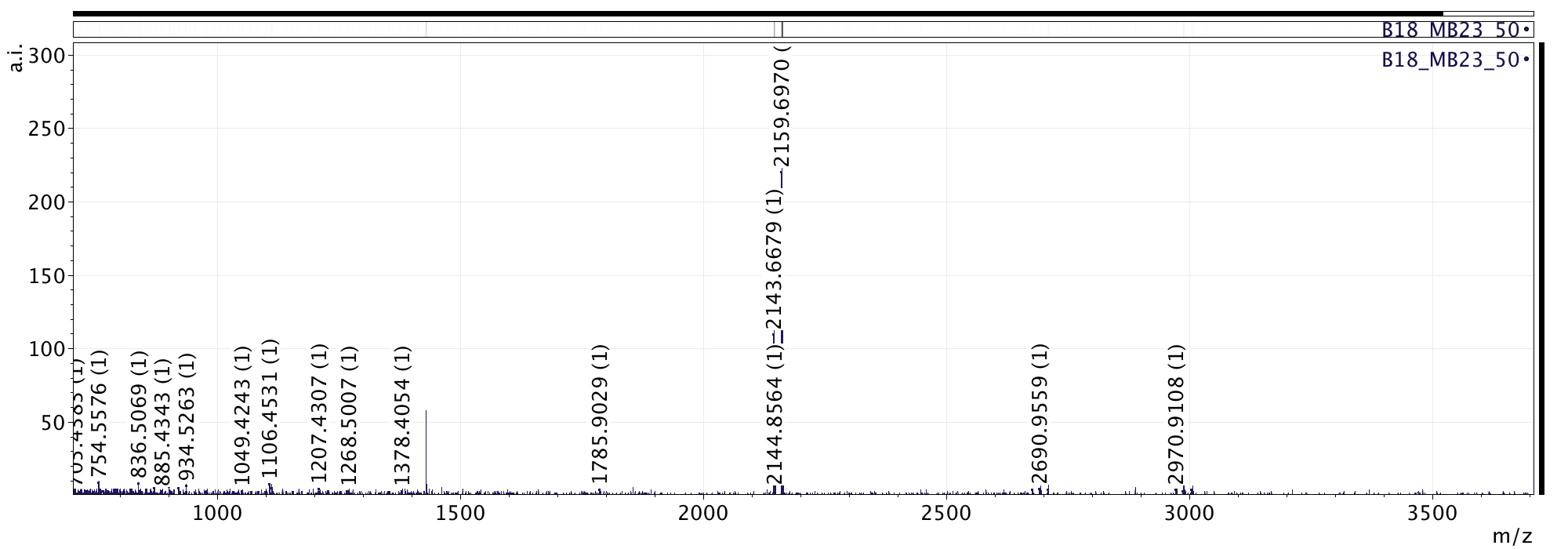 |
| #24  10% | 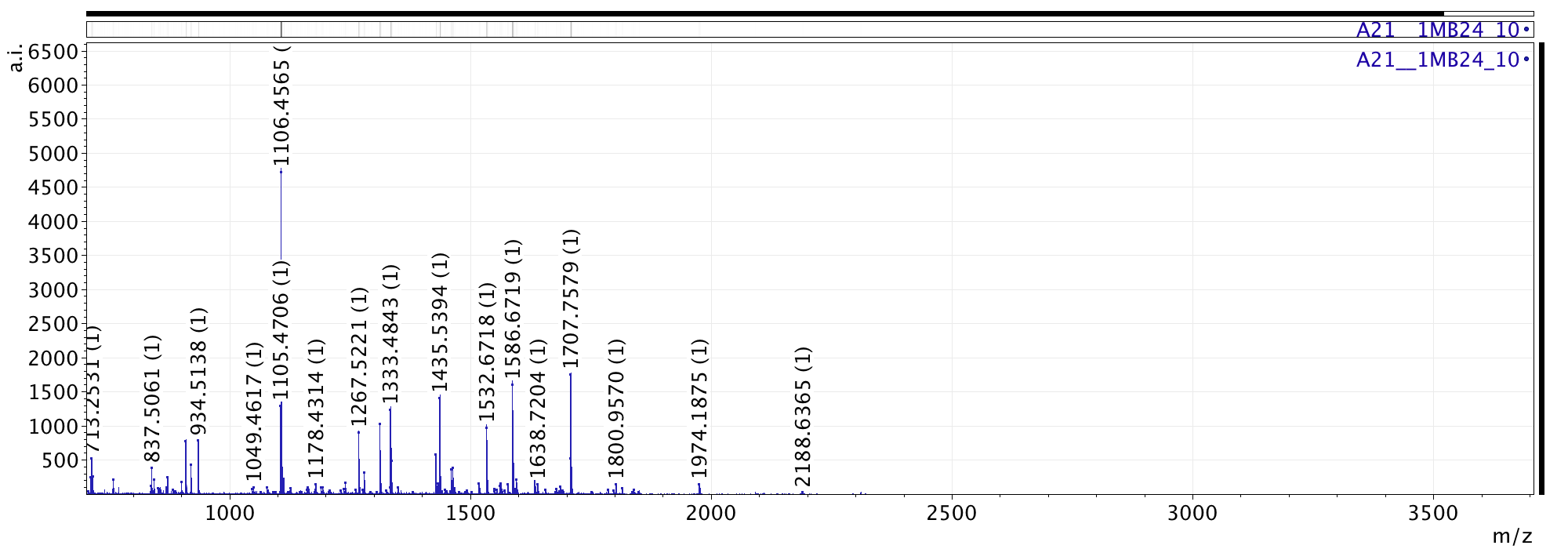 |
| #24  50% | 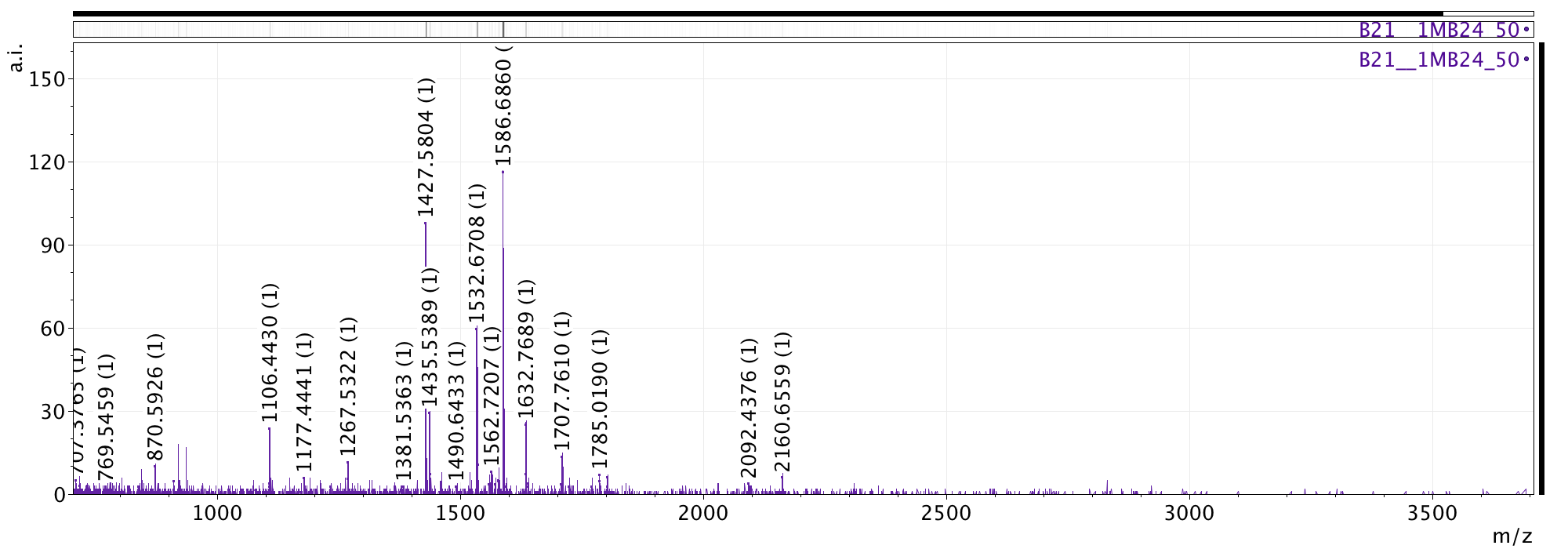 |
| #25  10% | 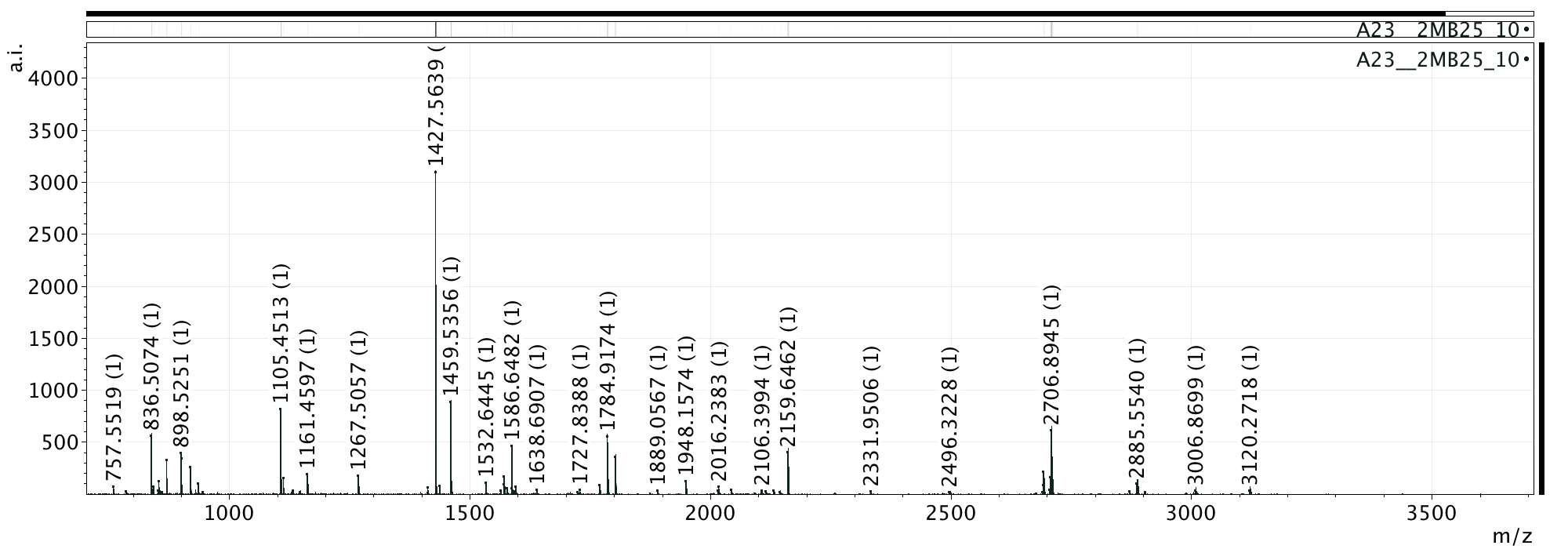 |
| #25  50% | 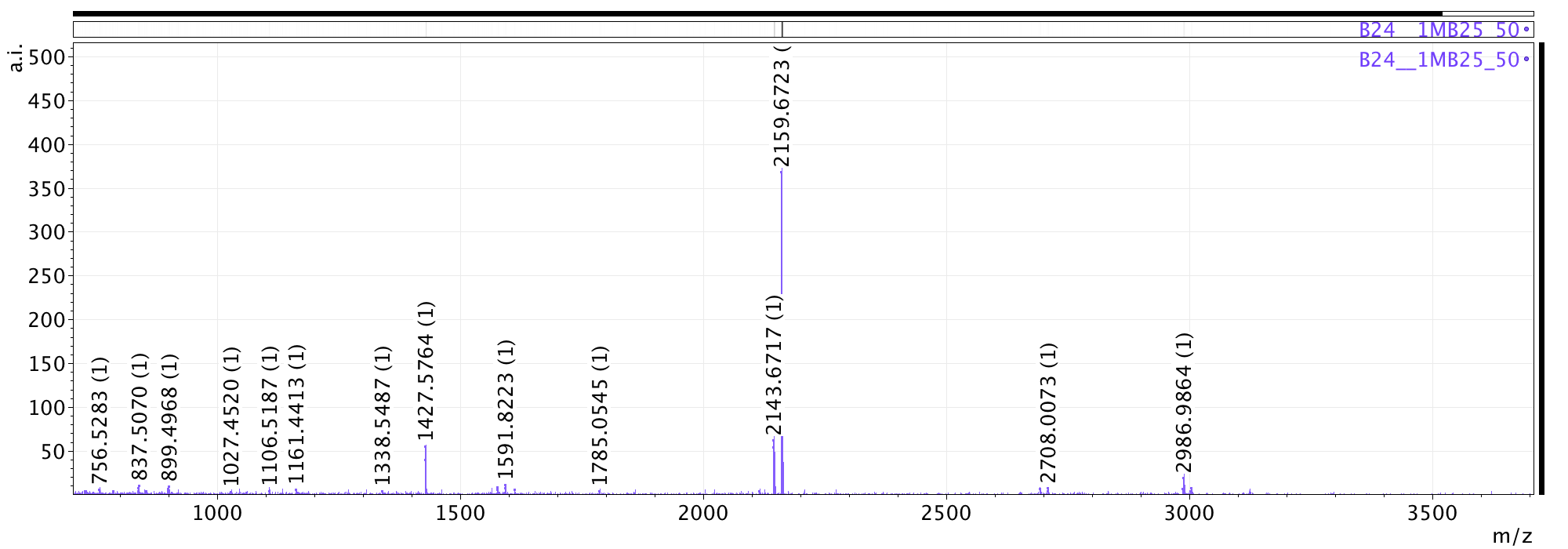 |
| #29  10% | 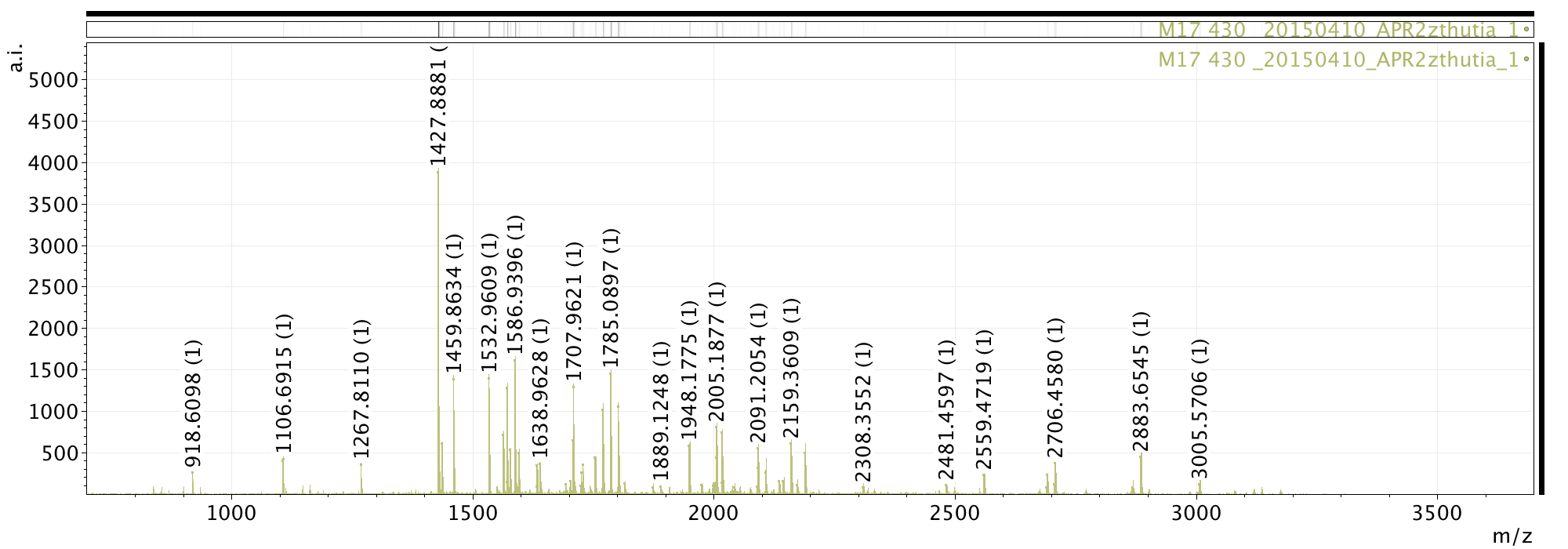 |
| #29 50% | 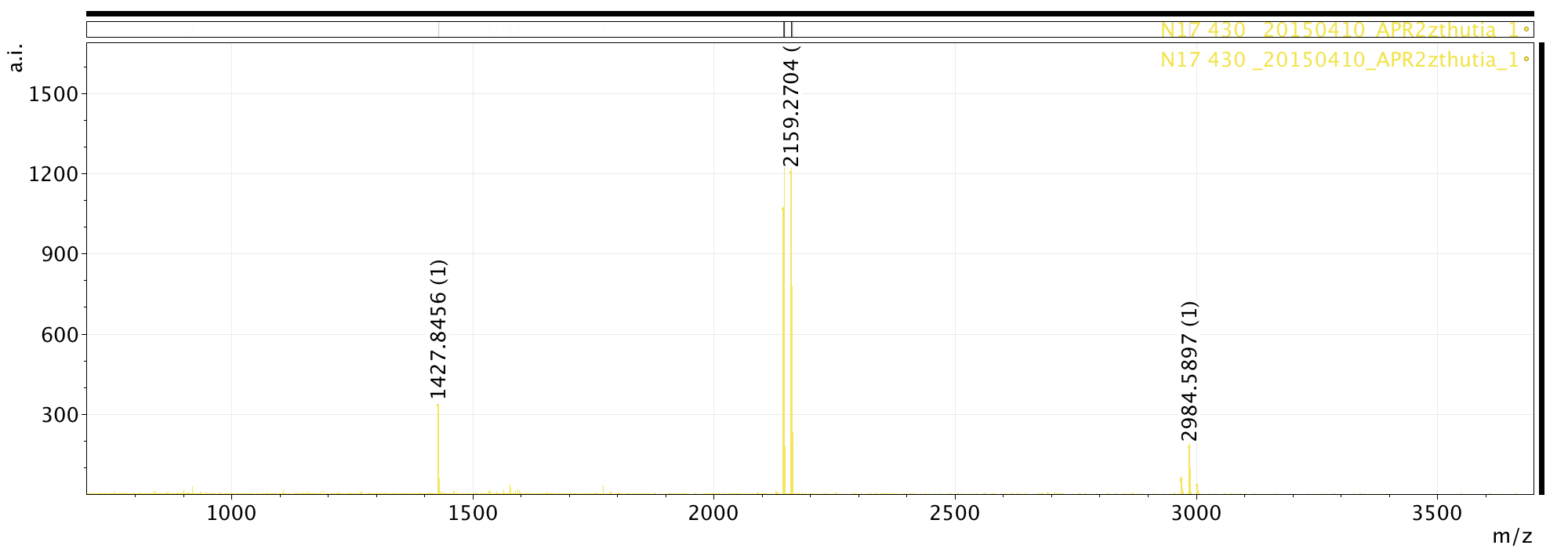 |
| #30  10% | 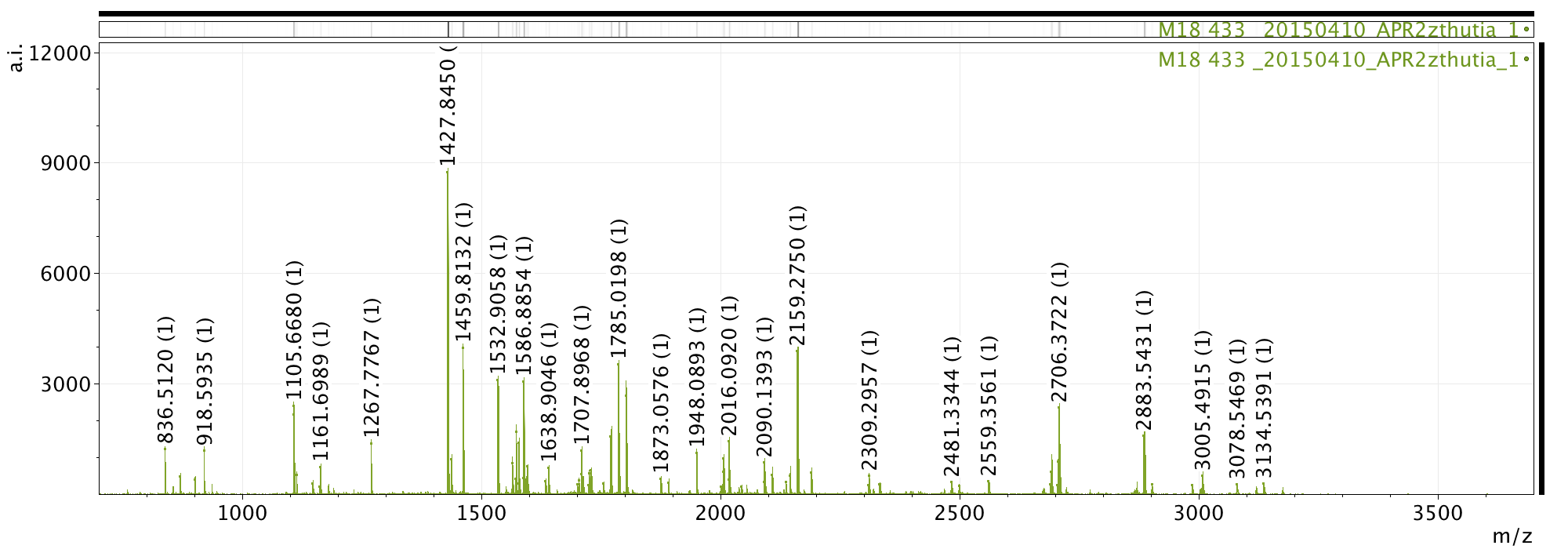 |
| #30  50% | 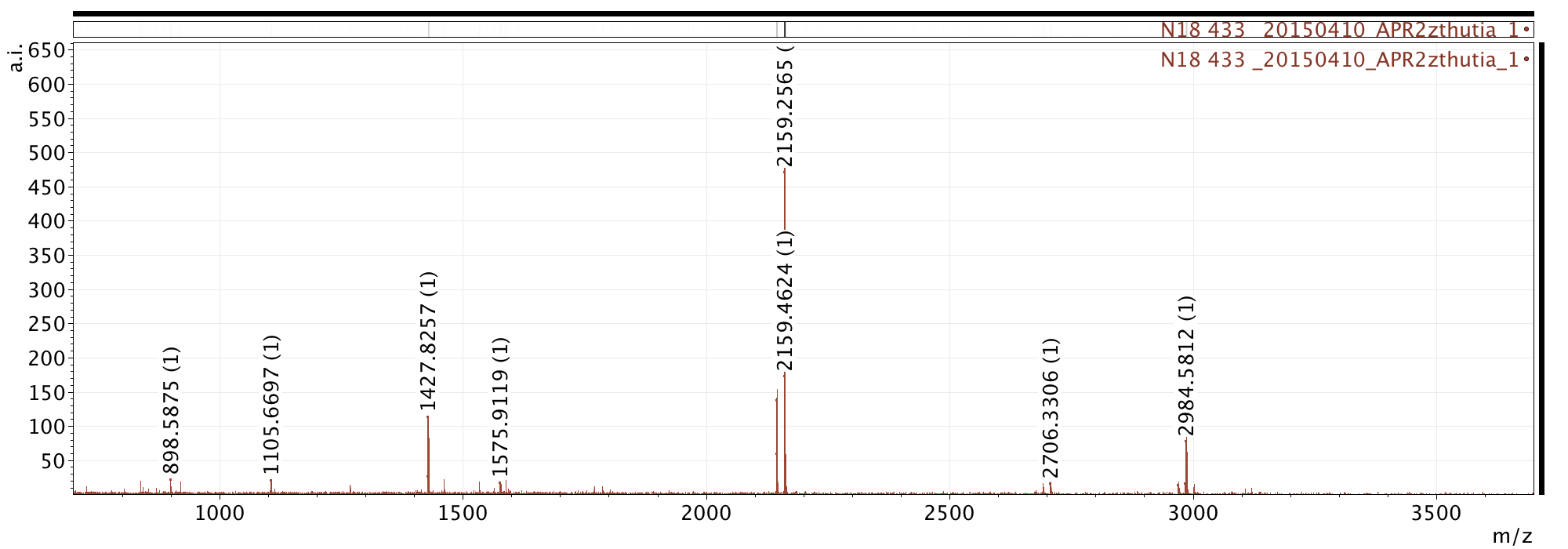 |
